# Functional specialization of retinal Müller cell endfeet depends on an interplay between two syntrophin isoforms

**DOI:** 10.1186/s13041-020-00581-w

**Published:** 2020-03-16

**Authors:** Shirin Katoozi, Shreyas B. Rao, Nadia Skauli, Stanley C. Froehner, Ole Petter Ottersen, Marvin E. Adams, Mahmood Amiry-Moghaddam

**Affiliations:** 1 Division of Anatomy, Department of Molecular Medicine, Institute of Basic Medical Sciences, University of Oslo, Post box 1105, Blindern, 0317 Oslo, Norway; 2 Department of Physiology and Biophysics, University of Washington, Seattle, WA 98195-7290 USA; 3 Present Address: President’s office, Karolinska Institutet, Nobels väg 6, 171 77 Stockholm, Sweden

**Keywords:** AQP4, β1-syntrophin, α1-syntrophin, Polarization, Retina, Müller cell

## Abstract

Retinal Müller cells are highly polarized macroglial cells with accumulation of the aquaporin-4 (AQP4) water channel and the inwardly rectifying potassium channel K_ir_4.1 at specialized endfoot membrane domains abutting microvessels and corpus vitreum. Proper water and potassium homeostasis in retina depends on these membrane specializations. Here we show that targeted deletion of β1-syntrophin leads to a partial loss of AQP4 from perivascular Müller cell endfeet and that a concomitant deletion of both α1- and β1-syntrophin causes a near complete loss of AQP4 from both perivascular and subvitreal endfoot membranes. α1-syntrophin is normally very weakly expressed in Müller cell endfeet but β1-syntrophin knockout mice display an increased amount of α1-syntrophin at these sites. We suggest that upregulation of perivascular α1-syntrophin restricts the effect of β1-syntrophin deletion. The present findings indicate that β1-syntrophin plays an important role in maintaining the functional polarity of Müller cells and that α1-syntrophin can partially substitute for β1-syntrophin in AQP4 anchoring. Functional polarization of Müller cells thus depends on an interplay between two syntrophin isoforms.

## Introduction

Macroglia are polarized cells whose functions are governed by distinct membrane domains that are specialized in terms of their structure, function and molecular composition [1–4]. Müller cells - a specialized class of retinal macroglia - have figured prominently in research on glial function and glial polarization [3]. Early studies on Müller cells revealed a strikingly uneven distribution of K^+^ conductance along the plasma membrane, with particularly high conductance in membrane domains facing vessels and corpus vitreum [5–12]. This finding laid the foundation for our current understanding of how macroglia removes excess K^+^ by siphoning and spatial buffering [13–15]. Later studies identified the biochemical substrate for this functional specialization. Thus it was found that Müller cell processes that abut on retinal vessels or corpus vitreum contain high concentrations of the inwardly rectifying K^+^ channel K_ir_4.1 [16–18]. Immunogold analyses showed that the very same endfoot domains also contain high densities of the water channel aquaporin-4 (AQP4) [16]. Taken together, functional and immunogold studies indicate that Müller cells - and macroglia in general - are endowed with specific membrane domains that are uniquely involved in K^+^ and water homeostasis [1, 4, 19, 20].

Immunoelectron microscopy revealed a loss of AQP4 from glial endfoot membranes in hippocampal specimens obtained from patients with mesial temporal lobe epilepsy [21]. More recent experimental observations indicate that loss of macroglial polarization occurs not only in epilepsy but also in stroke, edema and Alzheimer’s disease [22–26]. Indeed, loss of macroglial polarization may be a key pathophysiological event common to a wide range of neurological conditions. This begs the question of how macroglial polarization is established and maintained.

The first evidence that syntrophins are essential for glial polarization came with the study of Neely et al. [27], which indicated that AQP4 is linked to a dystrophin associated protein complex (DAP complex) through α1-syntrophin (α1-syn). In agreement, mice lacking α1-syn showed a loss of AQP4 from astrocyte endfeet in hippocampus and delayed clearance of K^+^ following high frequency synaptic activation [28]. A more recent study corroborates the idea that α1-syn is the single most important factor determining the size of the AQP4 pool in brain astrocytic endfeet [29]. However, deletion of α1-syn did not cause any significant loss of AQP4 from endfeet of retinal Müller cells [30], pointing to the existence of alternative anchoring mechanisms.

Here we exploit a newly generated transgenic mouse line [31] to investigate whether functional polarization of Müller cells depends on β1-syntrophin (β1-syn). We have previously used this mouse line to assess the role of β1-syn in anchoring of K_ir_4.1 [32]. The present study shows that β1-syn is the most important anchor of AQP4 in Müller cell processes but that α1-syn may partly substitute as anchor if β1-syn is lost. The unraveling of the mechanisms underlying Müller cell polarization opens new avenues for the understanding of glial function in normal brain and in disease.

## Materials and methods

### Animals

Age matched adult male (3- to 6- month old) mice with targeted deletion of the genes encoding α1-syn or β1-syn (α1-syn KO and β1-syn KO, respectively), or both genes (αβ1-syn KO, generated by crossing α1-syn KO and β1-syn KO mice) were used in this study. Mice of C57/BL6 background were used as wild type controls (WT). The details regarding generation and characterization of these mice are described previously [31, 33]. In addition, mice lacking the gene for AQP4 (AQP4 KO) were used as controls for antibody specificity [34]. Animals had access to food and water ad libitum. All the experimental procedures performed on mice were approved by the Institutional Animal Care and Use Committee of the University of Washington (#3298–02) and according to the European Council law on protection of laboratory animals, with the approval of the University of Oslo’s Animal Care and Use Committee (FOTS ID 8572). Every effort was made to minimize the number of animals.

### Perfusion and tissue preparation

Mice were anesthetized using isoflurane and then transcardially perfused with initial 20–30 s of ice cold 2% dextran in 0.1 M phosphate buffer (PB). The animals were perfusion fixed by using pH-shift protocol [35] where fixation was carried out using 4% formaldehyde and 0.2% picric acid in 0.1 M PB at pH 6.0 for 5 min, followed by the same fixative at pH 10.0 for 15 min. The eyes were dissected out, post fixed overnight and later stored in 1:10 dilution of the fixative in PB until further processing.

### Post embedding

Post embedding procedure was performed as previously described [36]. Briefly, small blocks of retina were dissected, cryoprotected in graded glycerol solution (10, 20 and 30% of glycerol in 0.1 M PB) and quickly frozen in propane cooled to − 170 °C in liquid nitrogen and subjected to freeze substitution. The samples were then embedded in methacrylate resin (Lowicryl HM20) and polymerized by UV light below 0 °C. Ultrathin sections of 80–100 nm were cut using Ultratome (Reichert Ultracut S, Leica) and placed on formvar carbon coated support film in Ni-grids.

### Immunogold electron microscopy

Immunogold labeling was performed as previously described [37]. Briefly, sections were rinsed in Tris-Buffered saline with Triton X-100 (TBST; 0.05% Tris-HCl, 0.9% NaCl and 0.1% Triton X-100), followed by the incubation with 2% (w/v) human serum albumin (HSA) at room temperature (RT) for 10 min. The sections were incubated with primary antibody overnight, followed by incubation with secondary goat anti-rabbit IgG antibody conjugated to 15 nm colloidal gold particle (1:20 dilution; Abcam; Cat#: ab27236; RRID:AB_954457) for 2 h. The sections were contrasted with 2% uranyl acetate and 0.3% lead citrate for 90 s each and examined using Tecnai 12 transmission electron microscope (FEI, Hillsboro, OR) at 80 kV.

Primary antibodies were: i) affinity-purified rabbit polyclonal antibody against AQP4 (1:400 dilution; Sigma Aldrich; Cat# A5971; RRID:AB_258270); ii) affinity-purified rabbit polyclonal antibody against α1-syn (SYN259; 1:100 dilution) which has previously been validated [38].

### Immunogold quantitation

Images (totaling ~ 1200) from outer plexiform layer (OPL), inner plexiform layer (IPL), ganglion cell layer (GCL), and subvitreous domain (Sub) of the retinae were acquired from each section and genotype. Care was taken to distinguish the different layers of the retina while taking the images at a magnification of 26,500x. Previous studies have shown an asymmetric distribution of AQP4 around the blood vessels of GCL [39]. In order to ensure proper sampling, images of vessels in the GCL were taken from the outer aspect of the retina where AQP4 was shown to be expressed. Quantification was performed as mean linear densities of gold particles by counting the gold particles within 23.5 nm of the inner leaflet of the membranes of interest [40, 41] using analySIS program (Soft Imaging Systems (SIS), Münster, Germany). In a separate experiment, AQP4 KO mice (*n* = 2) were used as controls for antibody specificity and the images of the vessels were acquired from OPL, IPL and GCL of retina.

### Immunofluorescence

For light microscopic immunofluorescence experiments, the perfusion fixed eyes were cryoprotected in gradient sucrose solution (10, 20 and 30% of sucrose in 0.1 M PB) before being frozen in OCT medium (Richard-Allan Scientific™ Neg-50™, Thermo Fisher Scientific; Cat#: 6502) using dry ice. Sections were cut at a thickness of 14 μm and were adhered on to glass slides and stored at − 80 °C until further use.

For immunofluorescence, the sections were thawed at RT, rinsed with phosphate buffer saline (PBS; 0.01 M) and were blocked using blocking solution (10% normal donkey serum, 1% bovine serum albumin (BSA; w/v), 0.5% triton in PBS) for 60 min. The sections were incubated overnight with affinity-purified rabbit polyclonal antibody against AQP4 (1:400 dilution; Sigma Aldrich; Cat# A5971; RRID:AB_258270) and mouse monoclonal antibody against Glutamine Synthetase (GS; 1:100 dilution; BD Biosciences; Cat# 610518, RRID:AB_397880) that was diluted in blocking solution with addition of sodium azide (0.01%). Following day, the sections were rinsed with PBS and then incubated with secondary antibodies diluted in blocking solution (Cy3 donkey-anti-rabbit; 1:500 dilution; Jackson ImmunoResearch Labs; Cat#: 711–165-152; RRID:AB_2307443 and Cy2 donkey-anti-mouse; 1:500 dilution; Jackson ImmunoResearch Labs; Cat#: 715–225-151; RRID:AB_2340827) for 1–2 h. Vessels were stained using DyLight® 649 conjugated tomato lectin (LEL, TL; Vector labs; Cat#: DL-1178). Nuclear staining was performed by incubating the sections with Hoechst 33258, (1:5000 dilution; Thermo Fisher Scientific; Cat#: H3569; RRID:AB_2651133) for 5 min and mounted using ProLong™ Gold Antifade Mountant (Thermo Fisher Scientific; Cat#: P36934; RRID:SCR_015961). Images were acquired using LSM 710 confocal microscope at 40x or 63x magnification, water objective (Carl Zeiss). For immunofluorescence experiments with α1-syn and β1-syn antibodies, eyeballs were removed from decapitated animals and quickly frozen in OCT media in a cryomold cassette in liquid nitrogen. Cryosections were cut at 14 μm thickness, adhered on to glass slides and stored at − 80 °C. Prior to staining, the sections were thawed to RT, fixed using 0.5% formaldehyde for 20 min and the staining procedure was continued as detailed above. The antibodies were diluted as follows: α1-syn (SYN259; 1:100 dilution) and β1-syn (Syn248; 1:100 dilution). These antibodies have previously been validated [38].

### Real time PCR

Detailed procedure for quantitative real-time PCR (qPCR) was performed as described in [32]. The primers used to detect *Aqp4* were; Forward (5′): TTTGGACCCGCAGTTATCAT; Reverse (3′): GTTGTCCTCCACCTCCATGT. TATA-box binding protein (*Tbp*) was used as the normalization gene. The primers used to detect *Tbp* were; Forward (5′): ACGGACAACTGCGTTGATTT; Reverse (3′): CAAGGCCTTCCAGCCTTATAG.

### Preparation of total protein lysates, SDS-PAGE and western blot

The detailed procedure for western blot analysis can be found as described in [32]. Briefly, 20 μg protein from WT, α1-syn KO and β1-syn KO male mouse retinae (*n* = 5 for each genotype) was separated on 10% Criterion™ 18-well TGX gels (BioRad, Hercules, CA, USA) at 160 V and wet blotted to PVDF membranes (BioRad) at 100 V for 45 min. Membranes were blocked in 5% BSA in 1X Tris-Buffered saline (TBS) for 1 h before overnight incubation with rabbit anti-aquaporin 4 (1:1000; Sigma-Aldrich; Cat# A5971; RRID:AB_258270), rabbit anti-α1-syn (1:1000; Abcam; Cat# ab11187; RRID:AB_2191794) or rabbit anti-β1-syn antibodies (Syn248; 1:1000 dilution) (Peters et al., 1997). Incubations with anti-HRP secondary antibodies (1:20000, Amersham, GE Healthcare; Cat# NA934, RRID:AB_772206) were performed for 1 h before TBST washes and detection of immunoreactive bands by SuperSignal™ West Pico Chemiluminescent Substrate (Thermo Fisher) on a BioRad Touch system. Subsequently, mouse anti-GAPDH (1:1000; Abcam; Cat# ab9484; RRID:AB_307274) was used as described for development of loading control bands.

### Statistical analysis

All the statistical analyses were carried out in SPSS (SPSS, Chicago, IL, USA). Sample sizes were determined based on previous studies [29, 32]. However, same number of animals were used for all the experiments. No data points or animals were excluded from any of the analyses.

For all western blot analyses (*n* = 5 for each genotype), bands were quantified as arbitrary background-subtracted density units in Image Studio Lite (Ver 5.2, Licor Biosciences, Nebraska, USA) and compared in SPSS Version 25 using independent samples t-test. Values are presented as percentage of the average wild-type values with mean values ± SD. * *p* < 0.05 was considered as significant.

For immunogold analyses, images were taken from four WT, four α1-syn KO, four β1-syn KO and four αβ1-syn KO mice. The data obtained from analySIS was transferred to SPSS Version 23 (SPSS, Chicago, IL) for statistical analysis. The researcher was blinded to the genotype of the animals during the entire procedure. Comparisons between groups were made by one-way ANOVA and post hoc Scheffé tests. Data are presented as mean ± SEM. * *p* < .05; ** *p* < .01; *** *p* < .001 were considered significant. Individual data points from each experiment is provided in ‘Additional file 2’.

## Results

### Targeted deletion of the gene encoding β1-syn does not affect transcript or protein level of AQP4

**Fig. 1:**
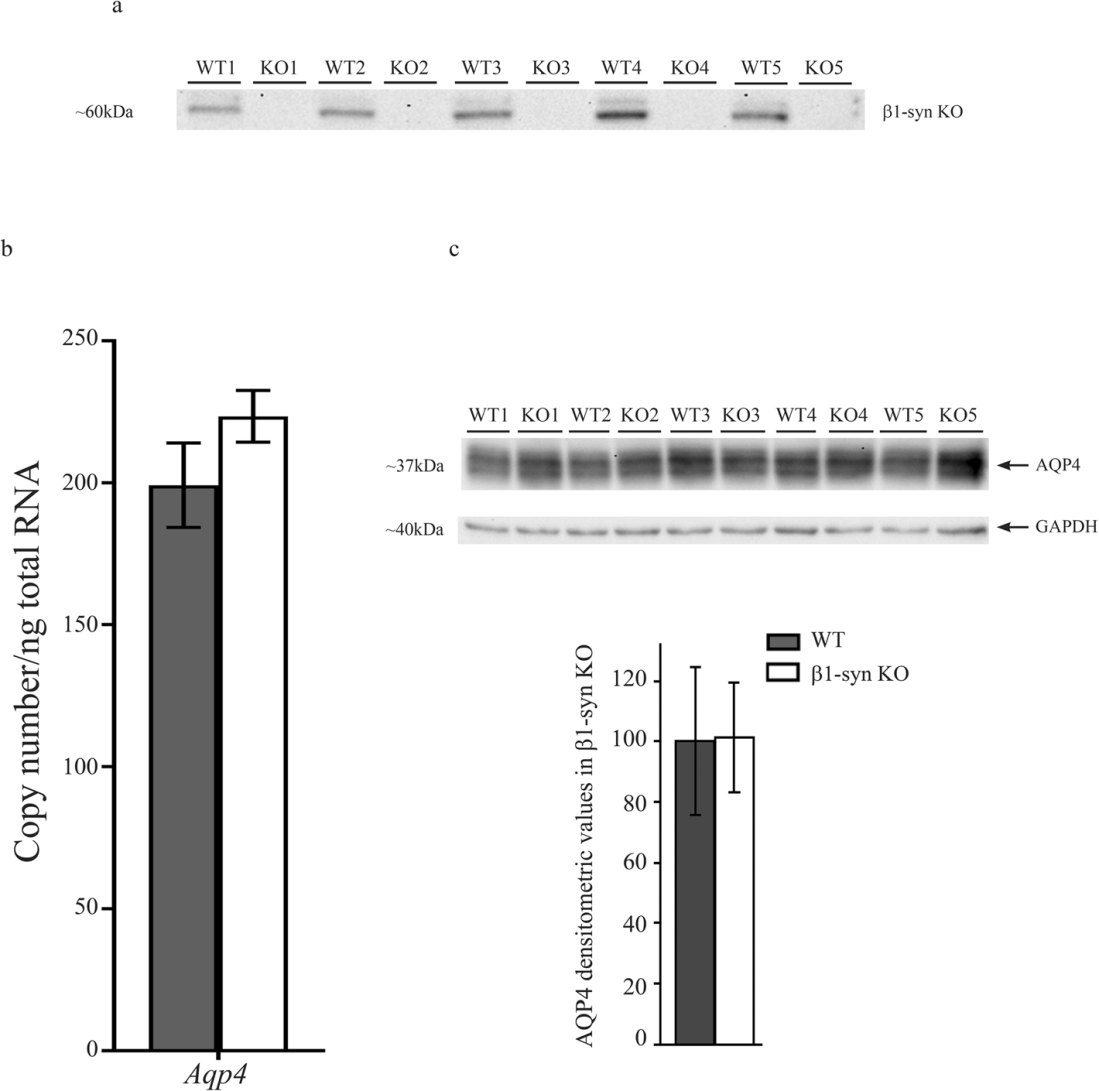
*Aqp4* expression in retinae of β1-syn KO mice. **a** Immunoblot from total protein lysates of WT and β1-syn KO retina samples. β1-syn KO mice lack an immunoreactive protein band at ~ 59 kDa corresponding to β1-syn, confirming the genotype. **b** qPCR analysis of total RNA extracted from eye using primers specific for *Aqp4*. No statistically significant difference in total *Aqp4* gene expression was observed between the two genotypes (*n* = 6 for WT and *n* = 7 for β1-syn KO). Statistics: Mann-Whitney *U*-test. *Tbp* was used as the normalization gene. Data shown as mean ± SEM. **c** Immunoblot showing AQP4 expression in total protein lysates from WT and β1-syn KO retinae. Statistical analysis of AQP4 expression in WT and β1-syn KO showed no difference in AQP4 protein expression between the two genotypes (*n* = 5 for each group). Densitometric values are expressed as percentage of average WT values ± SD. Statistics: independent samples t-test. GAPDH was used as the loading control

Western blot analysis on retinal protein lysates of β1-syn KO mice did not show any noticeable bands corresponding to the molecular weight of β1-syn (Fig. 1a). Successful knockout was also confirmed by qPCR and immunofluorescence [32]. The transcript level of *Aqp4* was not changed after β1-syn KO (Fig. 1b) and was also resistant to deletion of α1-syn, individually or in combination with deletion of β1-syn (Additional file 1: Fig. S1). Western blot analysis of retinae with antibodies to AQP4 did not reveal any difference between β1-syn KO mice and WT (Fig. 1c).

### Partial loss of perivascular AQP4 in mice with targeted deletion of β1-syn is accentuated by concomitant knockout of α1-syn

**Fig. 2:**
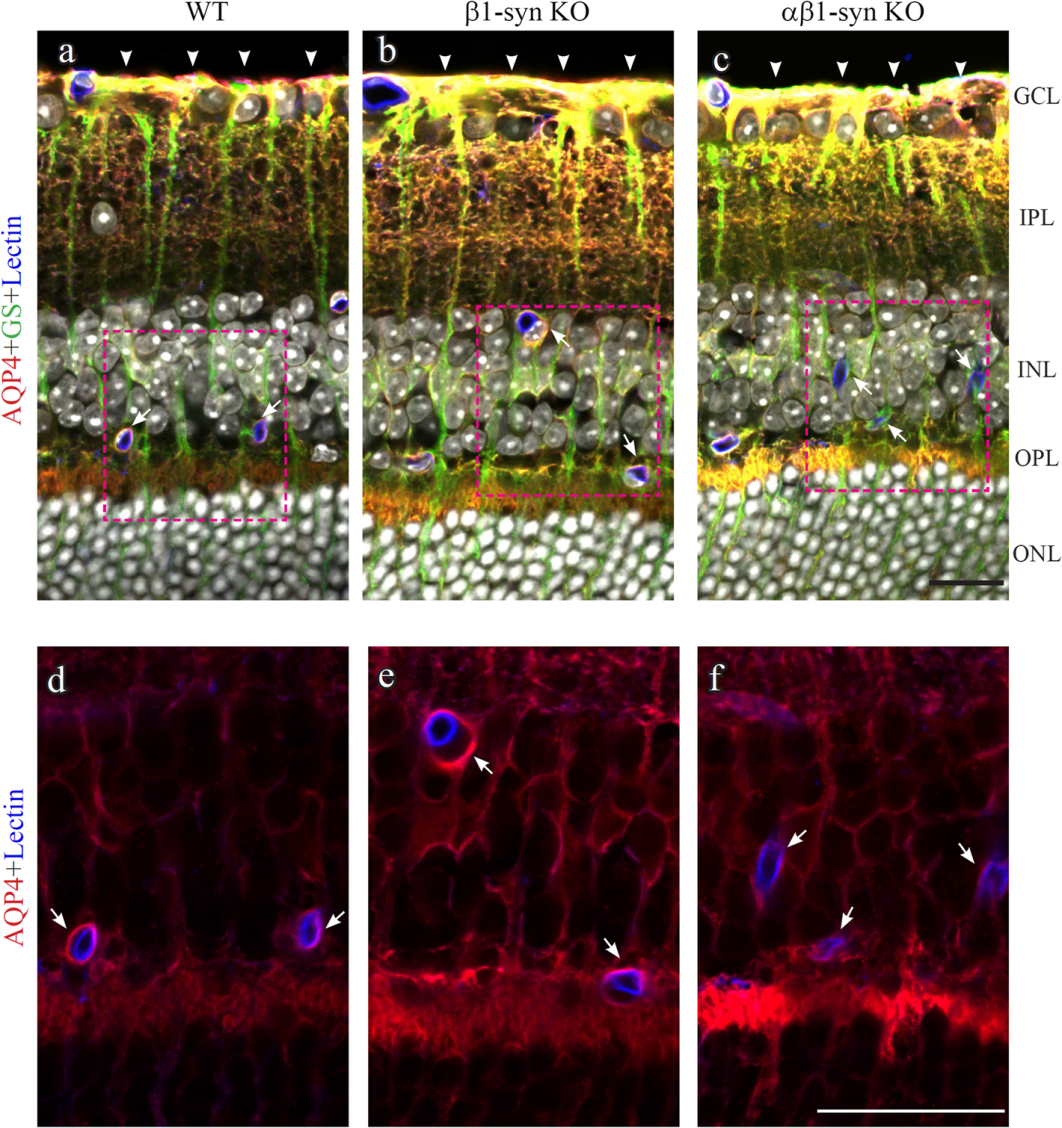
Immunofluorescence localization of AQP4 in retina. Confocal image showing AQP4 (in red), the Müller cell marker glutamine synthetase (GS; in green) and the endothelial marker lectin (in blue). AQP4 is concentrated in the perivascular (arrows) and in subvitreal membrane domains (arrowheads) in the WT (**a** and **d**) and β1-syn KO (**b** and **e**) animals. Perivascular AQP4 staining is strongly reduced in αβ1-syn KO mice (**c** and **f**). In both the knockouts, AQP4 labeling is increased along the entire length of Müller cell and around the inner nuclear layer. Nuclear staining is shown in white. GCL-ganglion cell layer; IPL-inner plexiform layer; INL-inner nuclear layer; OPL-outer plexiform layer; ONL-outer nuclear layer. Scale bars = 20 μm

The perivascular endfoot membrane plays a critical role in the exchange of water between blood and brain [28, 42]. Thus, we chose to focus on this membrane domain for further analysis with respect to AQP4 localization in retina. Our immunofluorescence analysis showed that targeted deletion of β1-syn produced an increased AQP4 labeling in retinal neuropil (Fig. 2b,e). This increase was even more pronounced in mice with concomitant knockout of α1- and β1-syntrophin (αβ1-syn KO) (Fig. 2c, f). In contrast, perivascular AQP4 staining was markedly reduced in the αβ1-syn KO mice (Fig. 2f). Whether perivascular labeling was attenuated also in β1-syn KO mice could not be resolved by immunofluorescence analysis (Fig. 2b, e). An immunogold analysis was required.

**Fig. 3:**
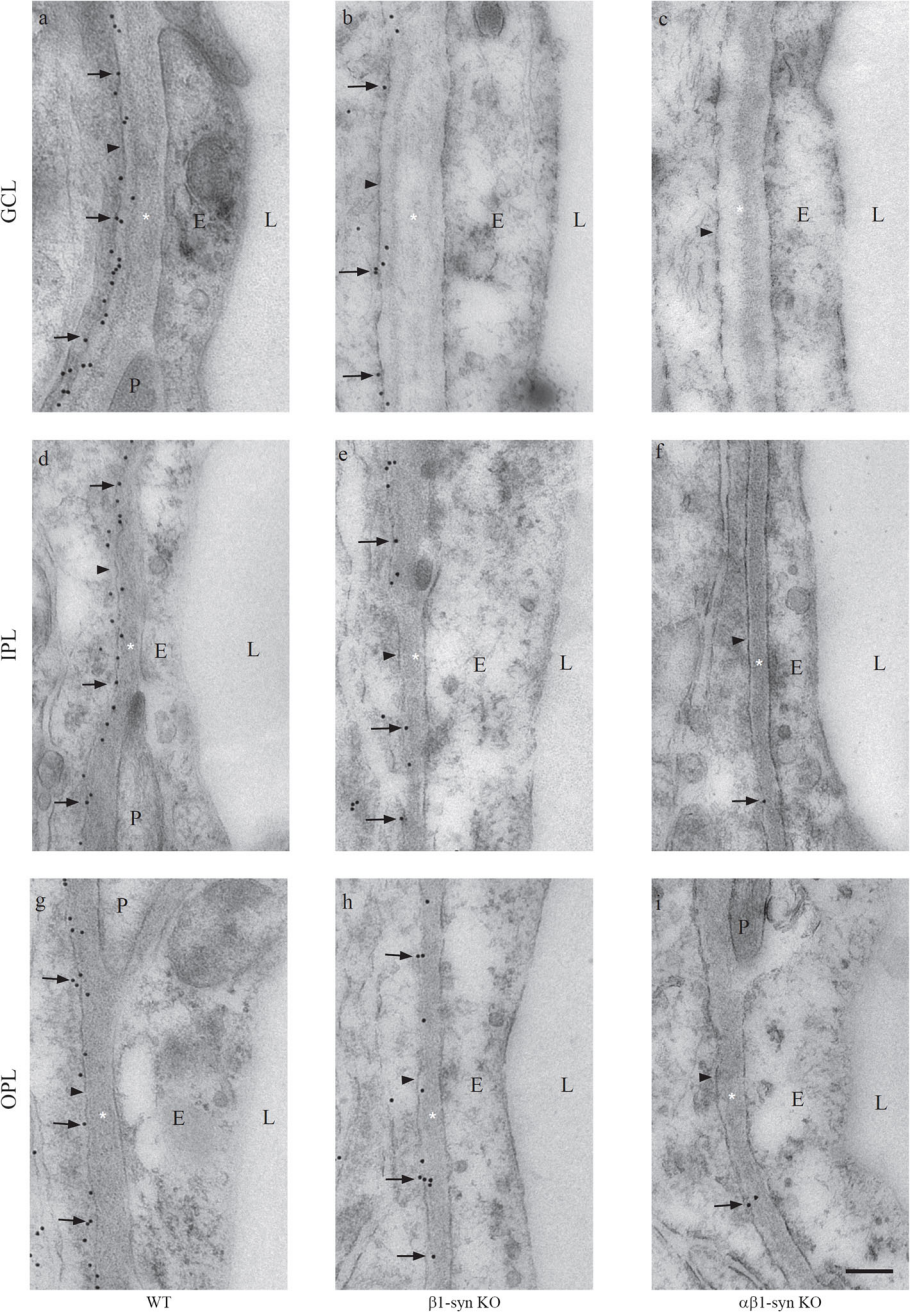
Electron micrographs showing AQP4 immunogold labeling in different vascular layers of the retina. The perivascular Müller cell membrane shows high density of immunogold labeling for AQP4 in WT retina (**a**, **d**, and **g**). Significant reduction in perivascular AQP4 labeling is seen in mice lacking β1-syn (**b**, **e** and **h**). A near complete loss of perivascular AQP4 labeling is seen in mice that lack both α1- and β1-syn (**c**, **f** and **i**). The arrowheads point to the endfoot domain facing the blood vessel. GCL-ganglion cell layer; IPL-inner plexiform layer; OPL-outer plexiform layer; L-lumen; E-endothelium; P-pericyte; * = basement membrane. Scale bar = 200 nm

**Fig. 4:**
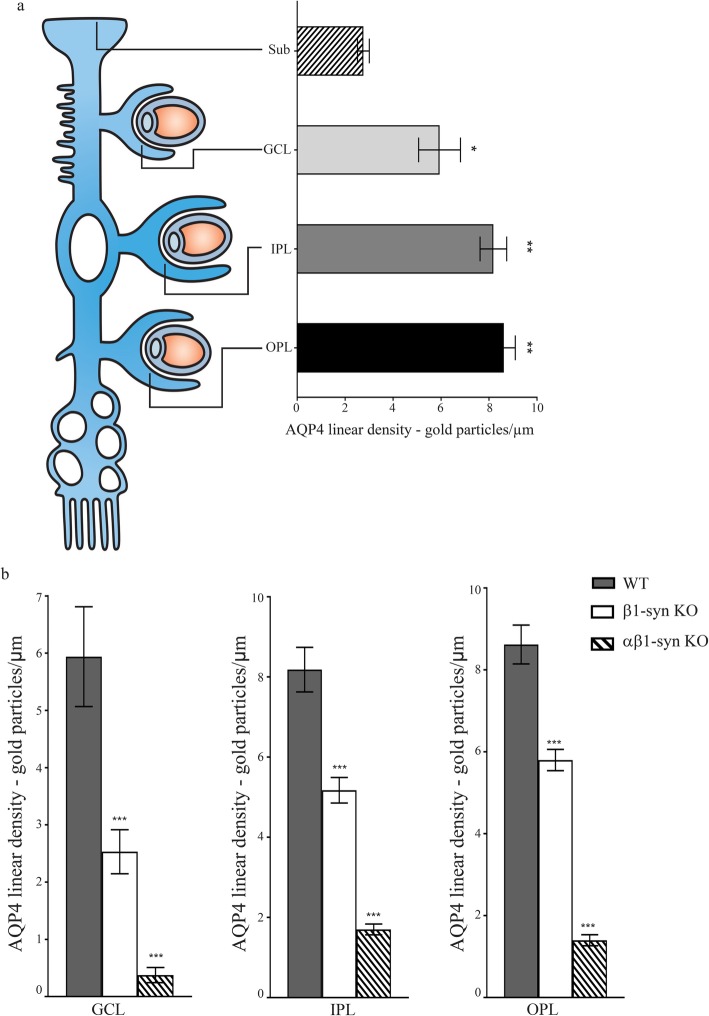
Quantitative analysis of the AQP4 immunogold labeling in different subregions of retina. **a** Schematic figure comparing the AQP4 distribution at the perivascular endfeet of the WT Müller cells in different layers i.e. outer plexiform layer (OPL), inner plexiform layer (IPL), ganglion cell layer (GCL) and the subvitreal endfeet (Sub). The AQP4 immunogold labeling (*n* = 4 mice) in Sub was significantly lower than GCL, IPL and OPL. **b** Quantitative analysis of AQP4 immunogold labeling in different retinal layers of WT, β1-syn KO and αβ1-syn KO mice. In mice lacking β1-syn, there was a significant reduction in the mean linear density of gold particles when compared with WT controls in all layers. In the double knockout mice, there was a near complete loss of perivascular labeling of AQP4 when compared with WT controls in all the layers (n = 4 for each genotype). Statistics: one-way ANOVA and post hoc Scheffé test. Data shown as mean ± SEM. * *p* < .05; ** *p* < .01; *** *p* < .001

In WT animals, immunogold particles signaling AQP4 were concentrated in perivascular endfoot membranes (Fig. 3a, d, g), consistent with the distinct and anatomically restricted immunofluorescence signal at this site. The linear density of AQP4 immunogold particles was highest in perivascular membranes of OPL and IPL and lowest in subvitreal endfoot membranes (Fig. 4a). Compared with WT, there was a noticeable reduction of AQP4 immunosignal in the perivascular endfoot membrane of β1-syn KO mice (Fig. 3b, e, h). In agreement with the immunofluorescence data, immunogold analysis showed a dramatic loss of AQP4 immunogold particles from perivascular Müller cell membranes of the αβ1-syn KO mice (Fig. 3c, f, i). Antibody specificity was confirmed by analysis of sections from AQP4 KO retina (Additional file 1: Fig. S2 and Fig. S3).

Quantitative analysis of the immunogold experiments showed that the loss of perivascular AQP4 depended on the retinal layer and ranged between ~ 32 and ~ 57% in β1-syn KO and between ~ 79 and ~ 94% in αβ1-syn KO mice compared to WT (Fig. 4b).

Since combined deletion of α1- and β1-syn results in near depletion of perivascular AQP4, we set out to quantify the specific contribution by α1-syn. Consistent with previous data [30], mice with targeted deletion of α1-syn did not differ significantly from WT animals in regard to the density of AQP4 in Müller cell endfeet (Additional file 1: Fig. S4a-d, g-i). The total level of AQP4 protein was unchanged after α1-syn KO (Additional file 1: Fig. S4e,f).

**Fig. 5:**
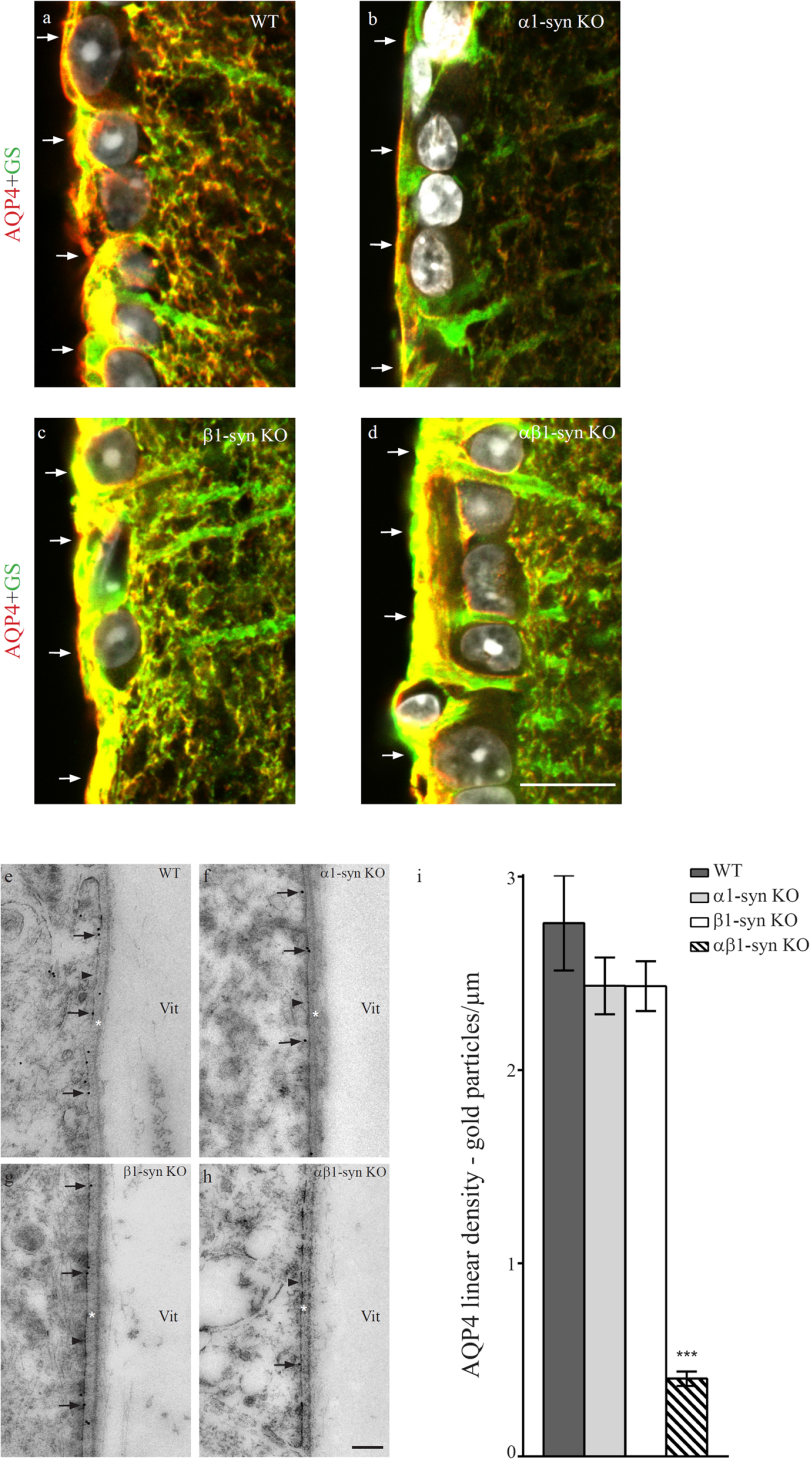
AQP4 localization requires the presence of both α1- and β1-syn in subvitreal domain. AQP4 (in red) is highly concentrated in the subvitreal membrane domains (arrows in **a**) in the WT animals. Deletion of either α1-syn (arrows in **b**) or β1-syn (arrows in **c**) alone does not affect AQP4 localization. Concomitant deletion of both α1- and β1-syn results in near complete loss of AQP4 (arrows in **d**). Nuclear staining is shown in white and Müller cell marker, glutamine synthetase (GS), is shown in green. Scale bar = 20 μm. Representative high resolution immunogold electron micrographs showing AQP4 localization in subvitreal domain. Concomitant deletion of both α1- and β1-syn results in near complete loss of AQP4 in subvitreal domain (**e** to **h**). Quantitative immunogold analysis shows a near complete loss of AQP4 in subvitreal domain (**i**; n = 4 for each group). Statistics: one-way ANOVA and post hoc Scheffé test. The arrowheads point to the endfoot domain facing the vitreous. Vit-vitreous body; * = basement membrane. Data shown as mean ± SEM. Scale bar = 200 nm. *** p < .001

Deletion of β1-syn or α1-syn individually had no effect on AQP4 labeling in the subvitreal endfoot membrane (Fig. 5b, c, f, g, i). However, immunofluorescence and immunogold signals for AQP4 were dramatically decreased in retinae of αβ1-syn KO mice (Fig. 5d, h, i; Additional file 1: Fig. S5).

### Loss of β1-syn results in increased expression of α1-syn

Since αβ1-syn KO led to a more dramatic loss of AQP4 than β1-syn KO, and since deletion of α1-syn alone did not result in any significant loss of perivascular AQP4, we asked whether deletion of β1-syn would lead to compensatory changes of α1-syn. The hypothesis would be that in the absence of β1-syn, α1-syn undergoes compensatory upregulation that helps tether a residual AQP4 pool to perivascular membranes. To test this, we performed western blot and quantitative immunogold analysis on WT and β1-syn KO retinae.

**Fig. 6:**
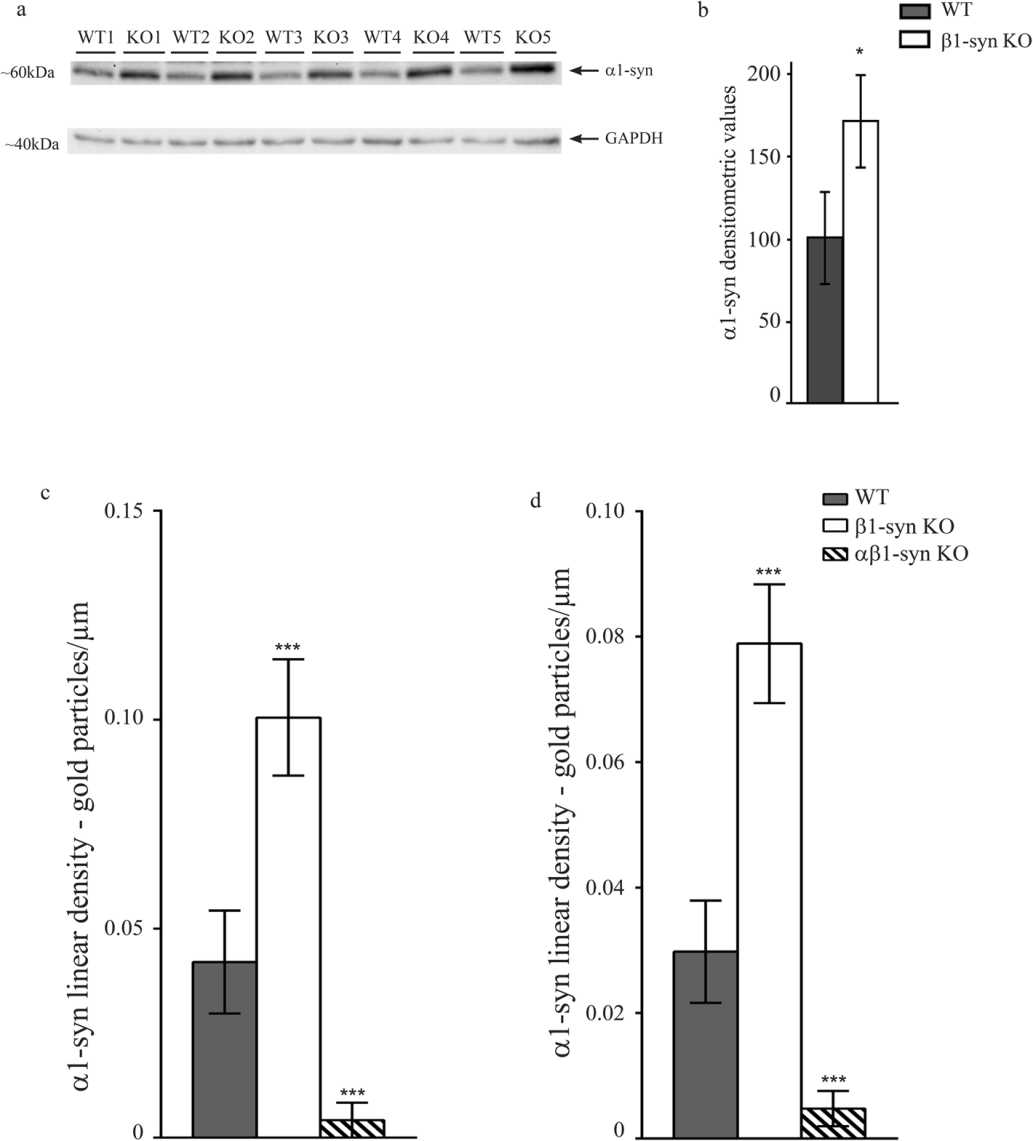
α1-syn protein level is increased in retinae of β1-syn KO mice. **a** Immunoblot showing α1-syn expression in total retinal protein lysates from WT and β1-syn KO retinae. Statistical analysis of α1-syn expression in WT and β1-syn KO shows that α1-syn protein level is significantly increased in β1-syn KO retina compared to WT (**b**; *n* = 5 for each group). Statistics: independent samples t-test. Densitometric values are expressed as percentage of average WT values ± SD. * *p* < .05. GAPDH was used as the loading control. Quantitative immunogold analysis showing α1-syn is increased in β1-syn KO retina, both in perivascular domain (**c**; n = 4 for each group) and in subvitreal domain (**d**; n = 4 for each group). Statistics: one-way ANOVA and post hoc Scheffé test. Data shown as mean ± SEM. *** p < .001

**Fig. 7:**
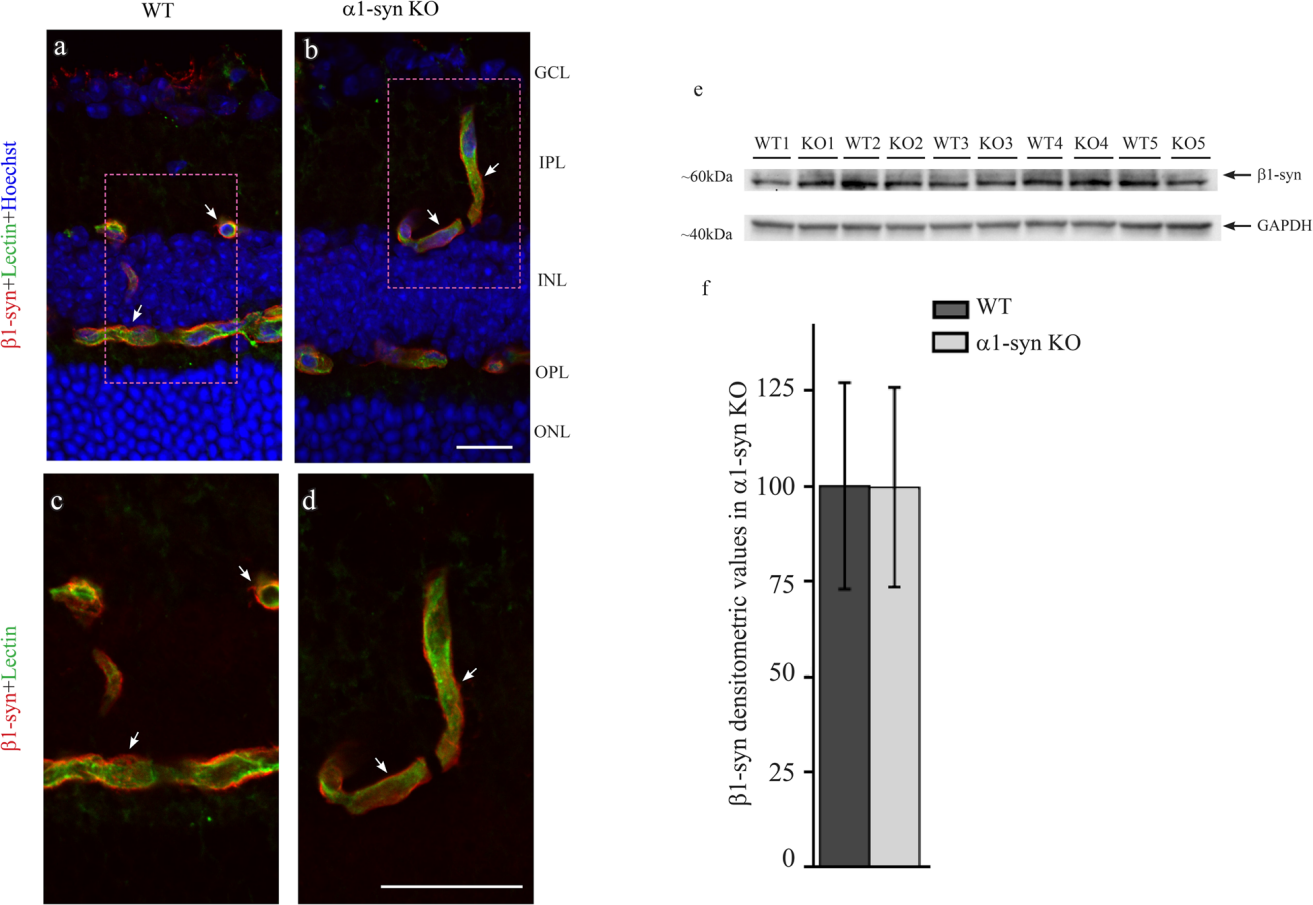
β1-syn protein level remains unchanged in retinae of α1-syn KO mice. Confocal images showing β1-syn (in red) immunofluorescence labeling in WT and in α1-syn KO mice along with the endothelial marker lectin (in green). β1-syn is concentrated at the perivascular region in WT animals (arrows in **a** and **c**). β1-syn labeling is not effected in mice lacking α1-syn (arrows in **b** and **d**). Nuclear staining is shown in blue. Immunoblot showing β1-syn expression in total protein lysates from WT and α1-syn KO retinae (**e**). Statistical analysis of β1-syn expression in WT and α1-syn KO showed no difference between the two genotypes (**f**; n = 5 for each group). Statistics: independent samples t-test. Densitometric values are expressed as percentage of average WT values ± SD. GAPDH was used as the loading control. GCL-ganglion cell layer; IPL-inner plexiform layer; INL-inner nuclear layer; OPL-outer plexiform layer; ONL-outer nuclear layer. Scale bars = 20 μm

Immunoblots revealed that β1-syn KO led to an increased expression of α1-syn (Fig. 6a, b). In agreement, perivascular and subvitreal immunogold labelling for α1-syn is significantly increased in β1-syn KO (Fig. 6c, d; Additional file 1: Fig. S6). Corroborating data were obtained by immunofluorescence analysis (Additional file 1: Fig. S7). In a previous study we found no change in α1-syn expression at mRNA level following targeted deletion of β1-syn [32]. Furthermore, western blot and immunofluorescence analyses from retinae of WT and α1-syn KO mice showed no difference in the level of β1-syn between the two genotypes (Fig. 7a-f). Thus, it appears that the upregulation of α1-syn in β1-syn KO mice occurs at the translational or post-translational level.

## Discussion

Müller cells are the archetypical class of macroglia for studies of homeostatic processes in the central nervous system. The realization that the anatomical polarization of macroglia has a functional and biochemical correlate stems in large measure from seminal studies of retinal Müller cells [5–9, 16, 22, 25, 39, 43, 44].

What are the mechanisms underlying the functional polarization that is so essential for glial physiology and pathophysiology? Obviously, the spatially restricted enrichment of specific membrane molecules must rely on distinct anchoring processes. The C-terminus of AQP4 provided a clue as to the nature of these processes. Thus, AQP4 contains the C-terminal -SSV sequence that is a putative recognition site of syntrophins - molecules that are integral parts of the dystrophin complex [27, 45–47]. It is now well established that this complex is responsible for the anchoring of AQP4 in brain astrocytes and that α1-syn is the immediate anchor for a major fraction of endfoot AQP4 in these cells [27–29, 46].

In the case of Müller cells, it remains to identify the anchoring molecules that dictate their functional polarization. Targeted deletion of α1-syn had only a modest effect on AQP4 expression in a mixed sample of retinal astrocytic and Müller cell processes [48] and no significant effect on AQP4 expression in perivascular Müller cell processes specifically sampled from the outer plexiform layer of the retina [30].

Here we provide evidence of an alternative anchoring mechanism for AQP4 that distinguishes Müller cells from brain macroglia. Specifically, we show that targeted deletion of β1-syn causes a partial loss of AQP4 from Müller cell endfeet and an almost complete loss if combined with a deletion of α1-syn. Double knockout mice retained less than 10% of their AQP4 pool in Müller cell endfeet. One would expect a residual AQP4 pool of this size even after a complete interruption of the specific anchoring mechanisms in endfoot membranes. Thus, AQP4 is found in the Müller cell membrane at large, at a density corresponding to 10% of that in perivascular membrane domains [39].

These findings, together with the unchanged levels of AQP4 protein and transcript levels in β1-syn KO mice retinae, confirm that the loss of AQP4 from the perivascular membranes is due to a mislocalization of AQP4 rather than an overall change in transcription or translation.

Mice with single knockout of α1-syn showed no significant reduction in AQP4 from Müller cell endfeet, thus confirming previous studies based on selective sampling of Müller cell processes. How then could deletion of α1-syn have such a strong additive effect when combined with the deletion of β1-syn? Judged by the faint immunohistochemical signal, α1-syn is weakly expressed in Müller cell endfoot membranes, thus explaining why single α1-syn KO mice retain their endfoot pool of AQP4. A key finding in the present study is that of a marked upregulation of α1-syn following β1-syn deletion. The most salient explanation of our findings is that this increase in α1-syn partially substitutes for the loss of β1-syn. Thus, only if both syntrophins are missing will the endfoot pool of AQP4 be removed.

The present study indicates that α1-syn partially compensates for a deletion of β1-syn but that β1-syn expression is resistant to α1-syn KO. The question remains whether there are additional syntrophins that should be considered in the context of Müller cell function. The retina shows an abundance of γ2-syntrophin (γ2-syn), as judged by our quantitative PCR data [32]. While γ2-syn knockout mice are not available as yet, the dramatic effect of the αβ1-syn double knockout on AQP4 polarization leaves little room for a contribution by γ2-syn. This is consistent with data suggesting that γ2-syn is expressed in neurons rather than in glia [48, 49]. As to β2-syn, this syntrophin has a distribution that is not consistent with a role in AQP4 anchoring and is predominantly found in the OPL [48]. Taken together, our data suggest that AQP4 anchoring in astrocytic endfoot membranes of retinal Müller cells mainly depends on expression of β1-syn and that α1-syn either partially (in perivascular membranes) or completely (in subvitreal membranes) compensates for β1-syn when the latter is knocked out. A compensatory upregulation of α1-syn was also observed in the skeletal muscle of β1-syn-KO mice [31].

In the present study we have used the well documented endfoot accumulation of AQP4 as a proxy for macroglial polarization. Our results suggest that the functional specialization of Müller cell membranes depends on an interplay between two syntrophin isoforms. While the effect of β1-syn knockout is partly compensated for by an upregulation of α1-syn, there is no change in β1-syn expression following targeted deletion of the gene encoding α1-syn. Previously, we showed that β1-syn is also responsible for the anchoring of a substantial fraction of the K_ir_4.1 pool in Müller cell endfeet [32]. There was no additive effect on K_ir_4.1 depletion in animals that lacked α1-syn as well as β1-syn. Taken together these studies suggest that β1-syn binds to K_ir_4.1 as well as AQP4 and that α1-syn couples less efficiently to the C-terminal end of K_ir_4.1 than to the C-terminal end of AQP4.

## Supplementary information

**Additional file 1 Figure S1** qPCR analysis of total RNA extracted from retinae using primers specific for *Aqp4*. No statistically significant difference in total *Aqp4* gene expression was observed between the WT, α1-syn KO and αβ1-syn KO mice (*n* = 4 for each group). Statistics: Mann-Whitney *U*-test. *Tbp* was used as the normalization gene. Data shown as mean ± SEM. **Figure S2** Control experiment for AQP4 antibody specificity. AQP4 antibody specificity was confirmed by incubating the retina samples from AQP4 KO and WT mice with the antibody. Confocal image showing a lack of specific labeling confirming the specificity of the antibody (panels a and b). GCL-ganglion cell layer; IPL-inner plexiform layer; INL-inner nuclear layer; OPL-outer plexiform layer; ONL-outer nuclear layer. Scale bars = 20 μm. Bottom: Immunogold staining also revealed a lack gold particles signaling AQP4 (bottom panel). Scale bar = 200 nm. **Figure S3** Electron micrographs from an AQP4 KO mouse retina section subjected to immunogold procedure using the anti-AQP4 antibody. There is no AQP4 immunogold labeling at the perivascular Müller cell endfeet in the three retinal vascular layers (a-c). GCL-ganglion cell layer; IPL-inner plexiform layer; OPL-outer plexiform layer; L-lumen; E-endothelium; P-pericyte; * = basement membrane. The arrowheads point to the endfoot domain facing the blood vessel. Scale bar = 1 μm. **Figure S4** Perivascular AQP4 localization is retained in retina of α1-syn KO mice. Confocal images showing AQP4 (in red) immunofluorescence labeling in WT and in α1-syn KO mice along with the endothelial marker lectin (in green). AQP4 is concentrated at the perivascular region in WT animals (arrows in panels a and c). AQP4 labeling is retained in mice lacking α1-syn (arrows in panels b and d). Nuclear staining is shown in blue. GCL-ganglion cell layer; IPL-inner plexiform layer; INL-inner nuclear layer; OPL-outer plexiform layer; ONL-outer nuclear layer. Scale bars = 20 μm. Immunoblot showing AQP4 expression in total protein lysates from WT and α1-syn KO retinae (panel e). Statistical analysis of AQP4 expression in WT and α1-syn KO showed no difference between the two genotypes (panel f; *n* = 5 for each group). Statistics: independent samples t-test. Densitometric values are expressed as percentage of average WT values ± SD. GAPDH was used as the loading control. High resolution electron micrographs showing perivascular labeling of AQP4 in WT and α1-syn KO mice (panels g and h). Quantitative immunogold analysis revealed no statistical difference between the two genotypes, in any of the layers (panel i; *n* = 4 for each group). Statistics: one-way ANOVA and post hoc Scheffé test. GCL-ganglion cell layer; IPL-inner plexiform layer; OPL-outer plexiform layer; L-lumen; E-endothelium; * = basement membrane. The arrowheads point to the endfoot domain facing the blood vessel. Data shown as mean ± SEM. Scale bar = 200 nm. **Figure S5** AQP4 localization in subvitreal domain of retinal Müller cells. AQP4 shown in red is highly concentrated in the subvitreal membrane domains (arrows in a and b) in the WT animals. Subvitreal localization of AQP4 is retained in mice that lack either α1-syn (arrows in c and d) or β1-syn (arrows in e and f). Deletion of both α1- and β1-syn results in near complete loss of AQP4 at the subvitreal Müller cell domains (arrows in g and h). Note a more distinct membrane staining of Müller cell stem processes in the αβ1-syn KO (arrowheads in g and h) compared to the other genotypes (arrowheads in a-f). Nuclei, mainly belonging to ganglion neurons, are visualized by nuclear labeling (grey and white in figures a, c, e and g). Glutamine synthetase (GS) is used as the marker of Müller cell cytosol and is shown in green. Scale bar = 20 μm. **Figure S6** Electron micrographs showing α1-syn labeling in perivascular Müller cell domains. Immunogold labeling of α1-syn is seen in retinae of WT (arrow in a) and β1-syn KO (arrows in c) mice. The immunogold particles are indicated by arrows. The labeling is very sparse but quantitative analysis of the immunogold labeling (main figure Fig. 6c and d) shows a significant difference between WT and β1-syn KO mice. Lack of immunogold particles is seen in α1-syn KO (b), and αβ1-syn KO (d) mice. The arrowheads point to the endfoot domain facing the blood vessel. L-lumen; E-endothelium; * = basement membrane. Scale bar = 200 nm. **Figure S7** Increased labeling of α1-syn in β1-syn KO retina. Confocal immunofluorescent images showing α1-syn (in red) labeling in retinae of WT (top panels) and β1-syn KO (bottom panels) mice. In mice lacking β1-syn, there was an increased labeling of α1-syn when compared to WT. Endothelial marker lectin is shown in green. Nuclear staining is shown in blue. Scale bars = 20 μm.

**Additional file 2.**

## Data Availability

All the datasets used and analyzed during this study are available from the corresponding author upon reasonable request.

## Abbreviations

AQP4: Aquaporin-4
BSA: Bovine serum albumin
DAP: Dystrophin associated protein
GCL: Ganglion cell layer
IPL: Inner plexiform layer
INL: Inner nuclear layer
OPL: Outer plexiform layer
ONL: Outer nuclear layer
Sub: Subvitreal
PB: Phosphate buffer
PBS: Phosphate buffered saline
HSA: Human serum albumin
GAPDH: Glyceraldehyde 3-phosphate dehydrogenase
TBP: TATA-box binding protein
RT: Room temperature
TBS: Tris buffered saline
WT: Wild type
KO: Knock-out
α1-syn: α1-syntrophin
β1-syn: β1-syntrophin
RRID: Research resource identifier (see scicrunch.org)

## References

[1] Amiry-Moghaddam M, Ottersen OP (2003). The molecular basis of water transport in the brain. Nat Rev Neurosci.

[2] Benfenati V, Ferroni S (2010). Water transport between CNS compartments: functional and molecular interactions between aquaporins and ion channels. Neuroscience.

[3] Reichenbach A, Bringmann A (2013). New functions of Muller cells. Glia.

[4] Verkhratsky A, Nedergaard M (2018). Physiology of Astroglia. Physiol Rev.

[5] Brew H, Gray PTA, Mobbs P, Attwell D (1986). Endfeet of retinal glial-cells have higher densities of ion channels that mediate K+ buffering. Nature.

[6] Newman EA (1984). Regional specialization of retinal glial cell membrane. Nature.

[7] Newman EA (1985). Membrane physiology of retinal glial (Muller) cells. J Neurosci.

[8] Newman EA (1987). Distribution of potassium conductance in mammalian Muller (glial) cells: a comparative study. J Neurosci.

[9] Newman EA (1993). Inward-rectifying potassium channels in retinal glial (Muller) cells. J Neurosci.

[10] Pannicke T, Uckermann O, Iandiev I, Wiedemann P, Reichenbach A, Bringmann A (2005). Ocular inflammation alters swelling and membrane characteristics of rat Muller glial cells. J Neuroimmunol.

[11] Pannicke T, Ivo Chao T, Reisenhofer M, Francke M, Reichenbach A (2017). Comparative electrophysiology of retinal Muller glial cells-a survey on vertebrate species. Glia.

[12] Connors NC, Kofuji P (2002). Dystrophin Dp71 is critical for the clustered localization of potassium channels in retinal glial cells. J Neurosci.

[13] Kofuji P, Connors NC (2003). Molecular substrates of potassium spatial buffering in glial cells. Mol Neurobiol.

[14] Kofuji P, Newman EA (2004). Potassium buffering in the central nervous system. Neuroscience.

[15] Butt AM, Kalsi A (2006). Inwardly rectifying potassium channels (Kir) in central nervous system glia: a special role for Kir4.1 in glial functions. J Cell Mol Med.

[16] Nagelhus EA, Horio Y, Inanobe A, Fujita A, Haug FM, Nielsen S, Kurachi Y, Ottersen OP (1999). Immunogold evidence suggests that coupling of K+ siphoning and water transport in rat retinal Muller cells is mediated by a coenrichment of Kir4.1 and AQP4 in specific membrane domains. Glia.

[17] Ishii M, Horio Y, Tada Y, Hibino H, Inanobe A, Ito M, Yamada M, Gotow T, Uchiyama Y, Kurachi Y (1997). Expression and clustered distribution of an inwardly rectifying potassium channel, KAB-2/Kir4.1, on mammalian retinal Muller cell membrane: their regulation by insulin and laminin signals. J Neurosci.

[18] Kofuji P, Ceelen P, Zahs KR, Surbeck LW, Lester HA, Newman EA (2000). Genetic inactivation of an inwardly rectifying potassium channel (Kir4.1 subunit) in mice: phenotypic impact in retina. J Neurosci.

[19] Nagelhus EA, Ottersen OP (2013). Physiological roles of aquaporin-4 in brain. Physiol Rev.

[20] Papadopoulos MC, Verkman AS (2013). Aquaporin water channels in the nervous system. Nat Rev Neurosci.

[21] Eid T, Lee TSW, Thomas MJ, Amiry-Moghaddam M, Bjornsen LP, Spencer DD, Agre P, Ottersen OP, de Lanerolle NC (2005). Loss of perivascular aquaporin 4 may underlie deficient water and K+ homeostasis in the human epileptogenic hippocampus. Proc Natl Acad Sci U S A.

[22] Iandiev I, Pannicke T, Biedermann B, Wiedemann P, Reichenbach A, Bringmann A (2006). Ischemia-reperfusion alters the immunolocalization of glial aquaporins in rat retina. Neurosci Lett.

[23] Yang J, Lunde LK, Nuntagij P, Oguchi T, Camassa LM, Nilsson LN, Lannfelt L, Xu Y, Amiry-Moghaddam M, Ottersen OP, Torp R (2011). Loss of astrocyte polarization in the tg-ArcSwe mouse model of Alzheimer's disease. J Alzheimers Dis.

[24] Alvestad S, Hammer J, Hoddevik EH, Skare O, Sonnewald U, Amiry-Moghaddam M, Ottersen OP (2013). Mislocalization of AQP4 precedes chronic seizures in the kainate model of temporal lobe epilepsy. Epilepsy Res.

[25] Pannicke T, Wurm A, Iandiev I, Hollborn M, Linnertz R, Binder DK, Kohen L, Wiedemann P, Steinhauser C, Reichenbach A, Bringmann A (2010). Deletion of aquaporin-4 renders retinal glial cells more susceptible to osmotic stress. J Neurosci Res.

[26] Smith AJ, Duan T, Verkman AS (2019). Aquaporin-4 reduces neuropathology in a mouse model of Alzheimer's disease by remodeling peri-plaque astrocyte structure. Acta Neuropathol Commun.

[27] Neely JD, Amiry-Moghaddam M, Ottersen OP, Froehner SC, Agre P, Adams ME (2001). Syntrophin-dependent expression and localization of Aquaporin-4 water channel protein. Proc Natl Acad Sci U S A.

[28] Amiry-Moghaddam M, Otsuka T, Hurn PD, Traystman RJ, Haug FM, Froehner SC, Adams ME, Neely JD, Agre P, Ottersen OPT, Bhardwaj A (2003). An alpha-syntrophin-dependent pool of AQP4 in astroglial end-feet confers bidirectional water flow between blood and brain. Proc Natl Acad Sci U S A.

[29] Hoddevik EH, Khan FH, Rahmani S, Ottersen OP, Boldt HB, Amiry-Moghaddam M (2017). Factors determining the density of AQP4 water channel molecules at the brain-blood interface. Brain Struct Funct.

[30] Enger R, Gundersen GA, Haj-Yasein NN, Eilert-Olsen M, Thoren AE, Vindedal GF, Petersen PH, Skare O, Nedergaard M, Ottersen OP, Nagelhus EA (2012). Molecular scaffolds underpinning macroglial polarization: an analysis of retinal Muller cells and brain astrocytes in mouse. Glia.

[31] Kim MJ, Whitehead NP, Bible KL, Adams ME, Froehner SC (2019). Mice lacking alpha-, beta1- and beta2-syntrophins exhibit diminished function and reduced dystrophin expression in both cardiac and skeletal muscle. Hum Mol Genet.

[32] Rao SB, Katoozi S, Skauli N, Froehner SC, Ottersen OP, Adams ME, Amiry-Moghaddam M (2019). Targeted deletion of beta1-syntrophin causes a loss of Kir 4.1 from Muller cell endfeet in mouse retina. Glia.

[33] Adams ME, Kramarcy N, Krall SP, Rossi SG, Rotundo RL, Sealock R, Froehner SC (2000). Absence of α-Syntrophin leads to structurally aberrant neuromuscular synapses deficient in Utrophin. J Cell Biol.

[34] Thrane AS, Rappold PM, Fujita T, Torres A, Bekar LK, Takano T, Peng W, Wang F, Rangroo Thrane V, Enger R (2011). Critical role of aquaporin-4 (AQP4) in astrocytic Ca2+ signaling events elicited by cerebral edema. Proc Natl Acad Sci U S A.

[35] Mathiisen TM, Lehre KP, Danbolt NC, Ottersen OP (2010). The perivascular astroglial sheath provides a complete covering of the brain microvessels: an electron microscopic 3D reconstruction. Glia.

[36] Prydz A, Stahl K, Puchades M, Davarpaneh N, Nadeem M, Ottersen OP, Gundersen V, Amiry-Moghaddam M (2017). Subcellular expression of aquaporin-4 in substantia nigra of normal and MPTP-treated mice. Neuroscience.

[37] Katoozi S, Skauli N, Rahmani S, Camassa LMA, Boldt HB, Ottersen OP, Amiry-Moghaddam M (2017). Targeted deletion of Aqp4 promotes the formation of astrocytic gap junctions. Brain Struct Funct.

[38] Peters MF, Adams ME, Froehner SC (1997). Differential association of syntrophin pairs with the dystrophin complex. J Cell Biol.

[39] Nagelhus EA, Veruki ML, Torp R, Haug F-M, Laake JH, Nielsen S, Agre P, Ottersen OP (1998). Aquaporin-4 Water Channel protein in the rat retina and optic nerve: polarized expression in Müller cells and fibrous astrocytes. J Neurosci.

[40] Amiry-Moghaddam M, Ottersen OP (2013). Immunogold cytochemistry in neuroscience. Nat Neurosci.

[41] Lunde LK, Camassa LM, Hoddevik EH, Khan FH, Ottersen OP, Boldt HB, Amiry-Moghaddam M (2015). Postnatal development of the molecular complex underlying astrocyte polarization. Brain Struct Funct.

[42] Frydenlund DS, Bhardwaj A, Otsuka T, Mylonakou MN, Yasumura T, Davidson KG, Zeynalov E, Skare O, Laake P, Haug FM (2006). Temporary loss of perivascular aquaporin-4 in neocortex after transient middle cerebral artery occlusion in mice. Proc Natl Acad Sci U S A.

[43] Dalloz C, Sarig R, Fort P, Yaffe D, Bordais A, Pannicke T, Grosche J, Mornet D, Reichenbach A, Sahel J (2003). Targeted inactivation of dystrophin gene product Dp71: phenotypic impact in mouse retina. Hum Mol Genet.

[44] Fort PE, Sene A, Pannicke T, Roux MJ, Forster V, Mornet D, Nudel U, Yaffe D, Reichenbach A, Sahel JA, Rendon A (2008). Kir4.1 and AQP4 associate with Dp71- and utrophin-DAPs complexes in specific and defined microdomains of Muller retinal glial cell membrane. Glia.

[45] Constantin B (1838). Dystrophin complex functions as a scaffold for signalling proteins. Biochim Biophys Acta.

[46] Amiry-Moghaddam M, Frydenlund DS, Ottersen OP (2004). Anchoring of aquaporin-4 in brain: molecular mechanisms and implications for the physiology and pathophysiology of water transport. Neuroscience.

[47] Blake DJ, Weir A, Newey SE, Davies KE (2002). Function and genetics of dystrophin and dystrophin-related proteins in muscle. Physiol Rev.

[48] Puwarawuttipanit W, Bragg AD, Frydenlund DS, Mylonakou MN, Nagelhus EA, Peters MF, Kotchabhakdi N, Adams ME, Froehner SC, Haug FM (2006). Differential effect of alpha-syntrophin knockout on aquaporin-4 and Kir4.1 expression in retinal macroglial cells in mice. Neuroscience.

[49] Piluso G, Mirabella M, Ricci E, Belsito A, Abbondanza C, Servidei S, Puca AA, Tonali P, Puca GA, Nigro V (2000). Gamma1- and gamma2-syntrophins, two novel dystrophin-binding proteins localized in neuronal cells. J Biol Chem.

