# Supplementary material for: Functional specialization of retinal Müller cell endfeet depends on an interplay between two syntrophin isoforms
Source: Mol Brain. 2020 Mar 16;13:40. doi: 10.1186/s13041-020-00581-w (PMC7074989; doi:10.1186/s13041-020-00581-w)

Functional specialization of retinal Müller cell endfeet depends on an interplay between two syntrophin isoforms

Shirin Katoozi[1 †], Shreyas B. Rao[1 †], Nadia Skauli[1], Stanley C. Froehner[2], Ole Petter Ottersen[1‡], Marvin E. Adams[2] and Mahmood Amiry-Moghaddam[1*]

[1]Division of Anatomy, Department of Molecular Medicine, Institute of Basic Medical Sciences, University of Oslo, Post box 1105, Blindern, 0317 Oslo, Norway

[2]Department of Physiology and Biophysics, University of Washington, Seattle, WA 98195-7290, USA

[†]Both authors contributed equally

[*]To whom correspondence should be addressed. Email: mahmo@medisin.uio.no

‡Current address: President's office, Karolinska Institutet, Nobels väg 6,171 77 Stockholm, Sweden

Number of Supplementary Figures: 7

Fig. S1 qPCR analysis of total RNA extracted from retinae using primers specific for *Aqp4*. No statistically significant difference in total *Aqp4* gene expression was observed between the WT, α1-syn KO and αβ1-syn KO mice (n=4 for each group). Statistics: Mann-Whitney *U*-test. *Tbp* was used as the normalization gene. Data shown as mean±SEM

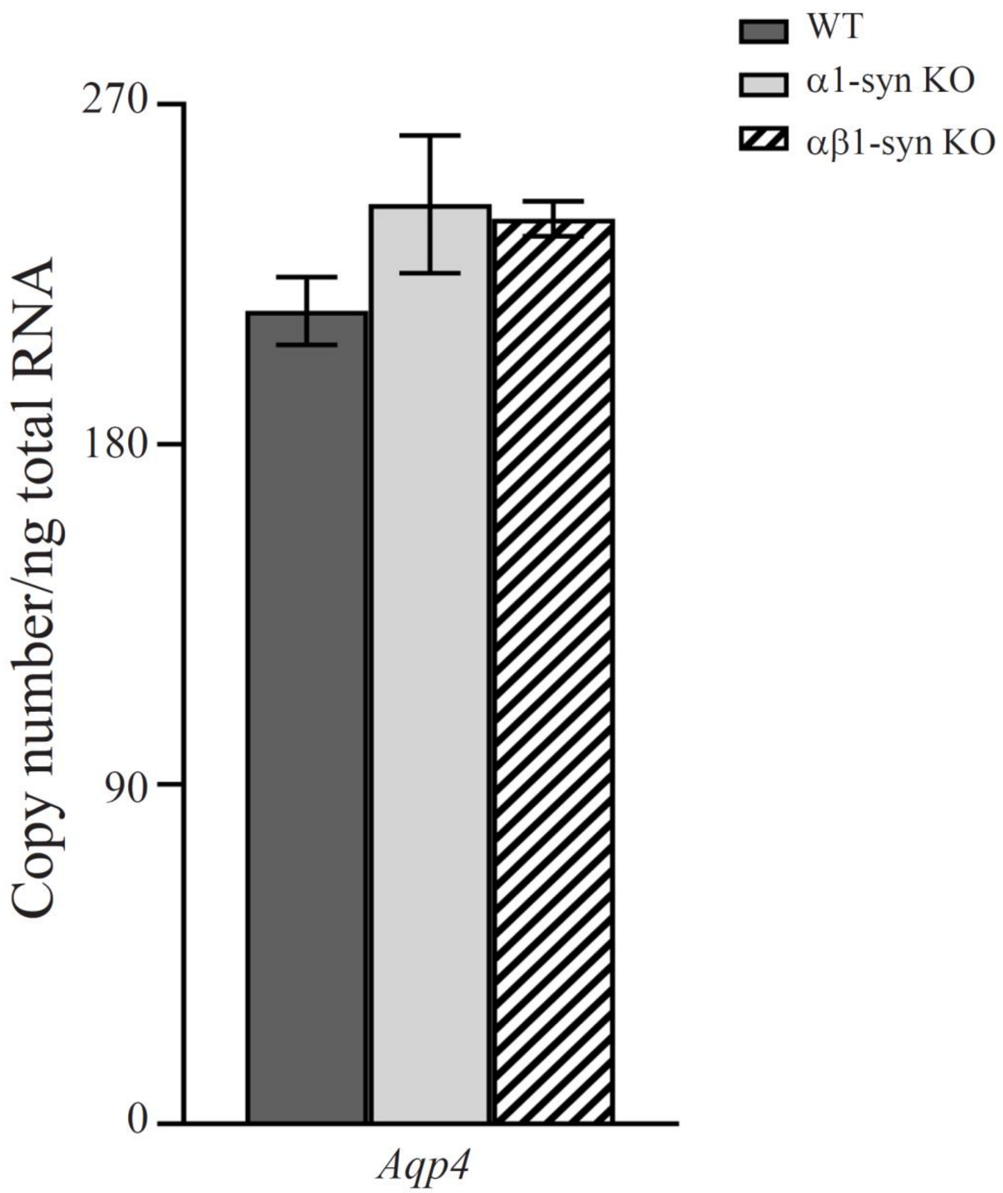

Fig. S2 Control experiment for AQP4 antibody specificity. AQP4 antibody specificity was confirmed by incubating the retina samples from AQP4 KO and WT mice with the antibody. Confocal image showing a lack of specific labeling confirming the specificity of the antibody (panels a and b). GCL-ganglion cell layer; IPL-inner plexiform layer; INL-inner nuclear layer; OPL-outer plexiform layer; ONL-outer nuclear layer. Scale bars=20 μm. Bottom: Immunogold staining also revealed a lack gold particles signaling AQP4 (bottom panel). Scale bar=200nm

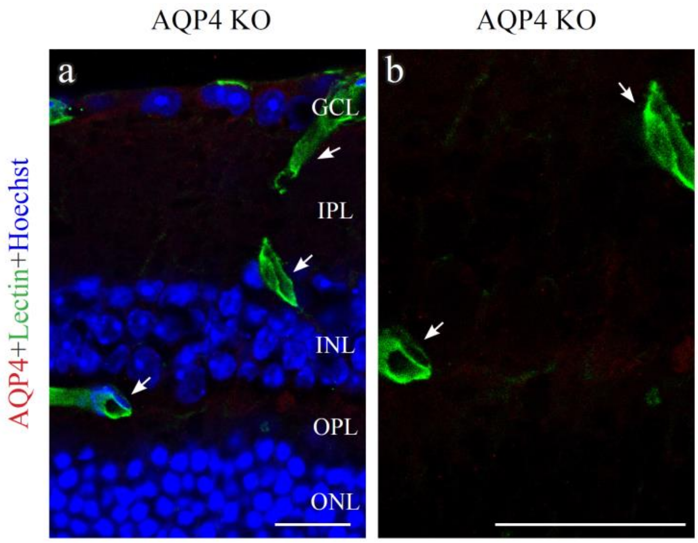

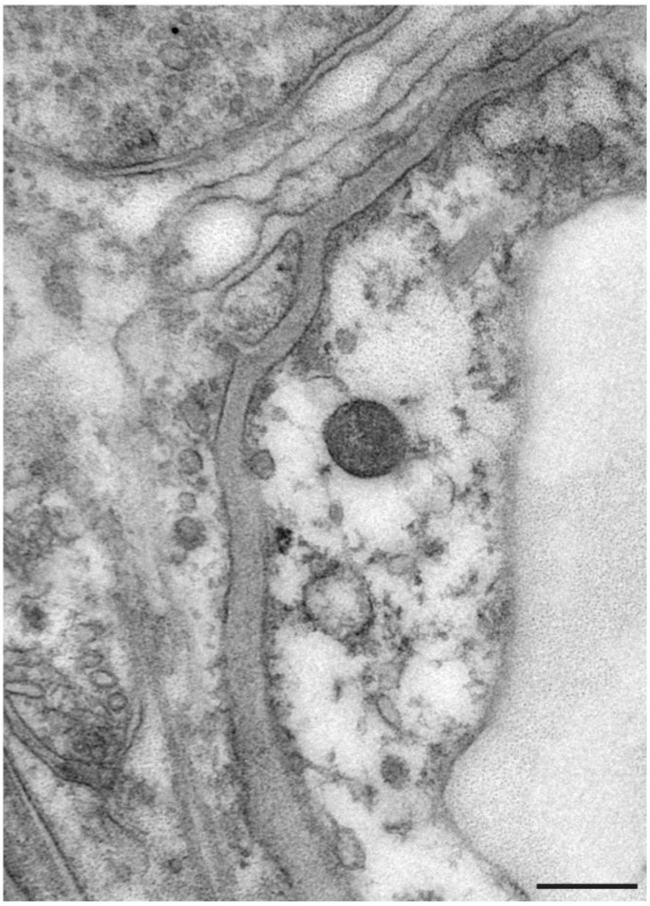

Fig. S3 Electron micrographs from an AQP4 KO mouse retina section subjected to immunogold procedure using the anti-AQP4 antibody. There is no AQP4 immunogold labeling at the perivascular Müller cell endfeet in the three retinal vascular layers (a-c). GCL-ganglion cell layer; IPL-inner plexiform layer; OPL-outer plexiform layer; L-lumen; E-endothelium; P-pericyte; *=basement membrane. The arrowheads point to the endfoot domain facing the blood vessel. Scale bar=1 μm

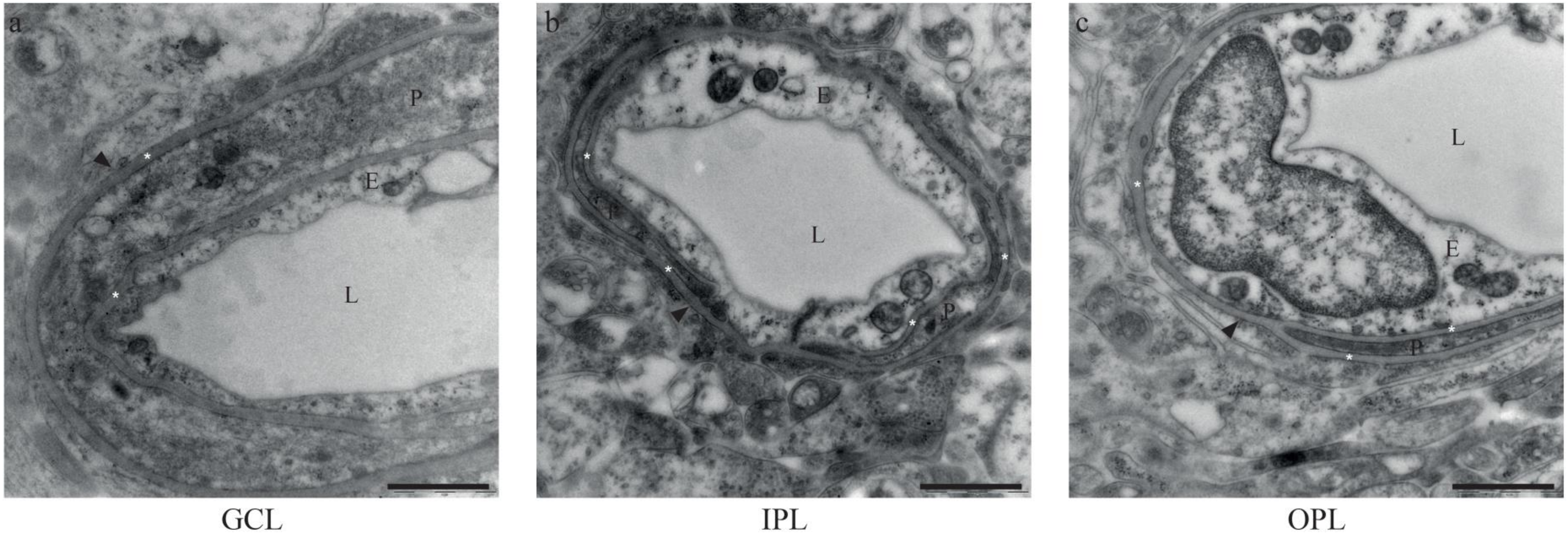

Fig. S4 Perivascular AQP4 localization is retained in retina of α1-syn KO mice. Confocal images showing AQP4 (in red) immunofluorescence labeling in WT and in α1-syn KO mice along with the endothelial marker lectin (in green). AQP4 is concentrated at the perivascular region in WT animals (arrows in panels a and c). AQP4 labeling is retained in mice lacking α1-syn (arrows in panels b and d). Nuclear staining is shown in blue. GCL-ganglion cell layer; IPL-inner plexiform layer; INL-inner nuclear layer; OPL-outer plexiform layer; ONL-outer nuclear layer. Scale bars=20μm. Immunoblot showing AQP4 expression in total protein lysates from WT and α1-syn KO retinae (panel e). Statistical analysis of AQP4 expression in WT and α1-syn KO showed no difference between the two genotypes (panel f; n=5 for each group). Statistics: independent samples t-test. Densitometric values are expressed as percentage of average WT values ± SD. GAPDH was used as the loading control. High resolution electron micrographs showing perivascular labeling of AQP4 in WT and α1-syn KO mice (panels g and h). Quantitative immunogold analysis revealed no statistical difference between the two genotypes, in any of the layers (panel i; n=4 for each group). Statistics: one-way ANOVA and post hoc Scheffé test. GCL-ganglion cell layer; IPL-inner plexiform layer; OPL-outer plexiform layer; L-lumen; E-endothelium; *=basement membrane. The arrowheads point to the endfoot domain facing the blood vessel. Data shown as mean±SEM. Scale bar=200nm

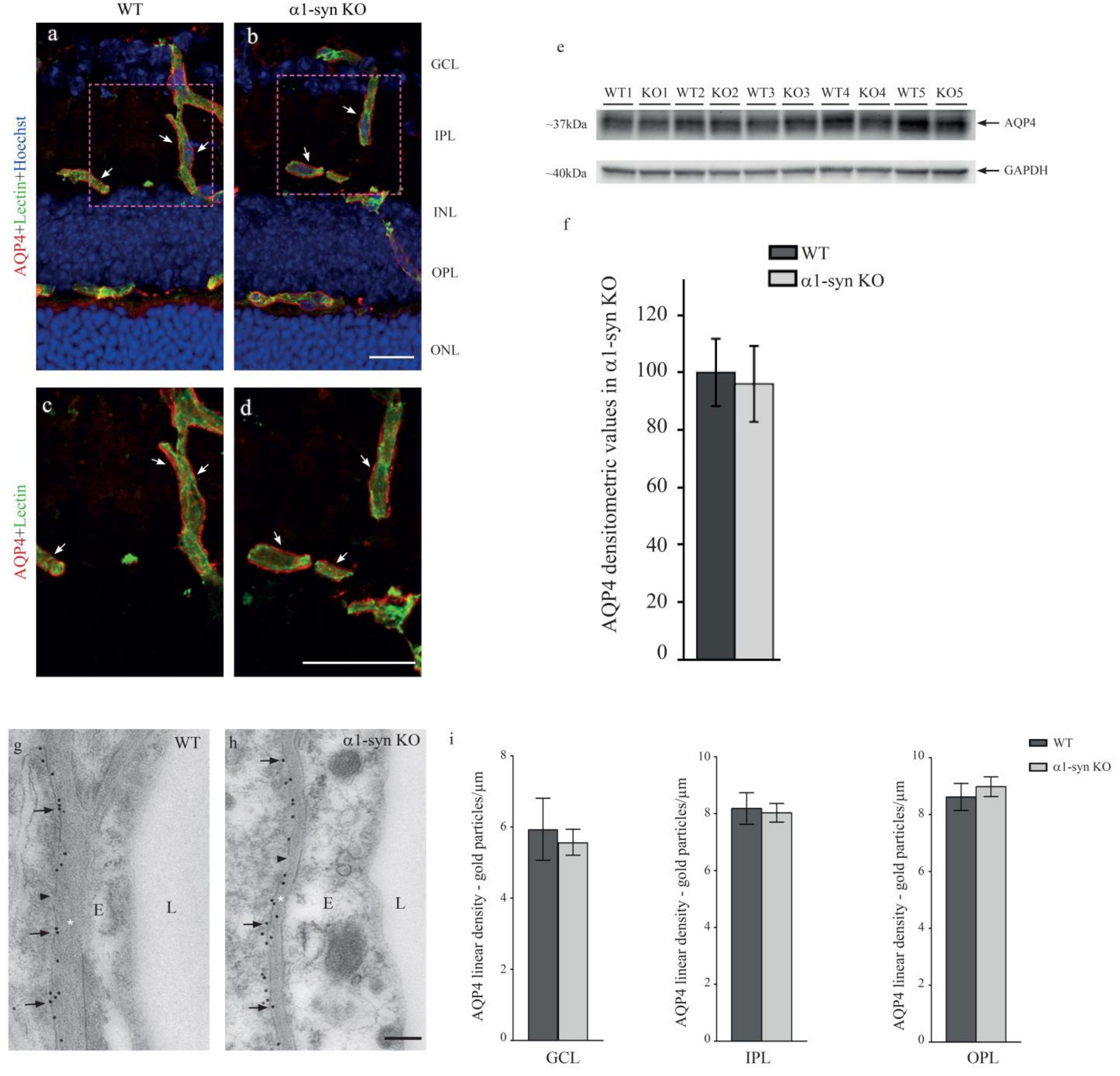
WT
α1-syn KO
a
b
c
d
e
f
g
h
i
AQP4+Lectin+Hoechst
AQP4+Lectin
GCL
IPL
INL
OPL
ONL
WT1 KO1 WT2 KO2 WT3 KO3 WT4 KO4 WT5 KO5
~37kDa
~40kDa
AQP4
GAPDH
WT
α1-syn KO
AQP4 densitometric values in α1-syn KO
0
20
40
60
80
100
120
E
L
AQP4 linear density - gold particles/μm
GCL
IPL
OPL

Fig. S5 AQP4 localization in subvitreal domain of retinal Müller cells. AQP4 shown in red is highly concentrated in the subvitreal membrane domains (arrows in a and b) in the WT animals. Subvitreal localization of AQP4 is retained in mice that lack either α1-syn (arrows in c and d) or β1-syn (arrows in e and f). Deletion of both α1- and β1-syn results in near complete loss of AQP4 at the subvitreal Müller cell domains (arrows in g and h). Note a more distinct membrane staining of Müller cell stem processes in the αβ1-syn KO (arrowheads in g and h) compared to the other genotypes (arrowheads in a-f). Nuclei, mainly belonging to ganglion neurons, are visualized by nuclear labeling (grey and white in figures a, c, e and g). Glutamine synthetase (GS) is used as the marker of Müller cell cytosol and is shown in green. Scale bar=20µm

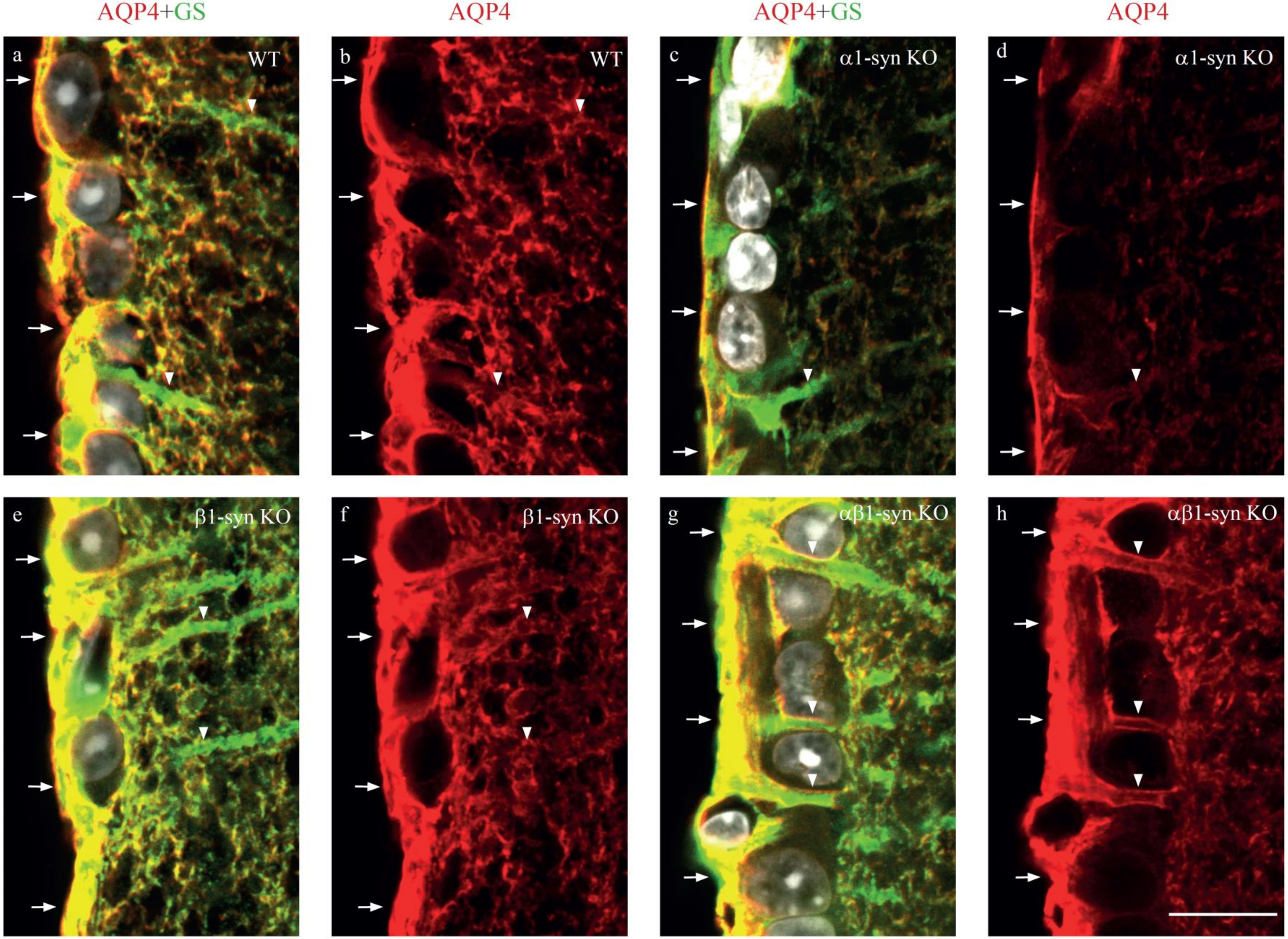
AQP4+GS
AQP4
AQP4+GS
AQP4
a
WT
b
WT
c
α1-syn KO
d
α1-syn KO
e
β1-syn KO
f
β1-syn KO
g
αβ1-syn KO
h
αβ1-syn KO

Fig. S6 Electron micrographs showing α1-syn labeling in perivascular Müller cell domains. Immunogold labeling of α1-syn is seen in retinae of WT (arrow in a) and β1-syn KO (arrows in c) mice. The immunogold particles are indicated by arrows. The labeling is very sparse but quantitative analysis of the immunogold labeling (main figure Fig. 6c and d) shows a significant difference between WT and β1-syn KO mice. Lack of immunogold particles is seen in α1-syn KO (b), and αβ1-syn KO (d) mice. The arrowheads point to the endfoot domain facing the blood vessel. L-lumen; E-endothelium; *=basement membrane. Scale bar=200 nm

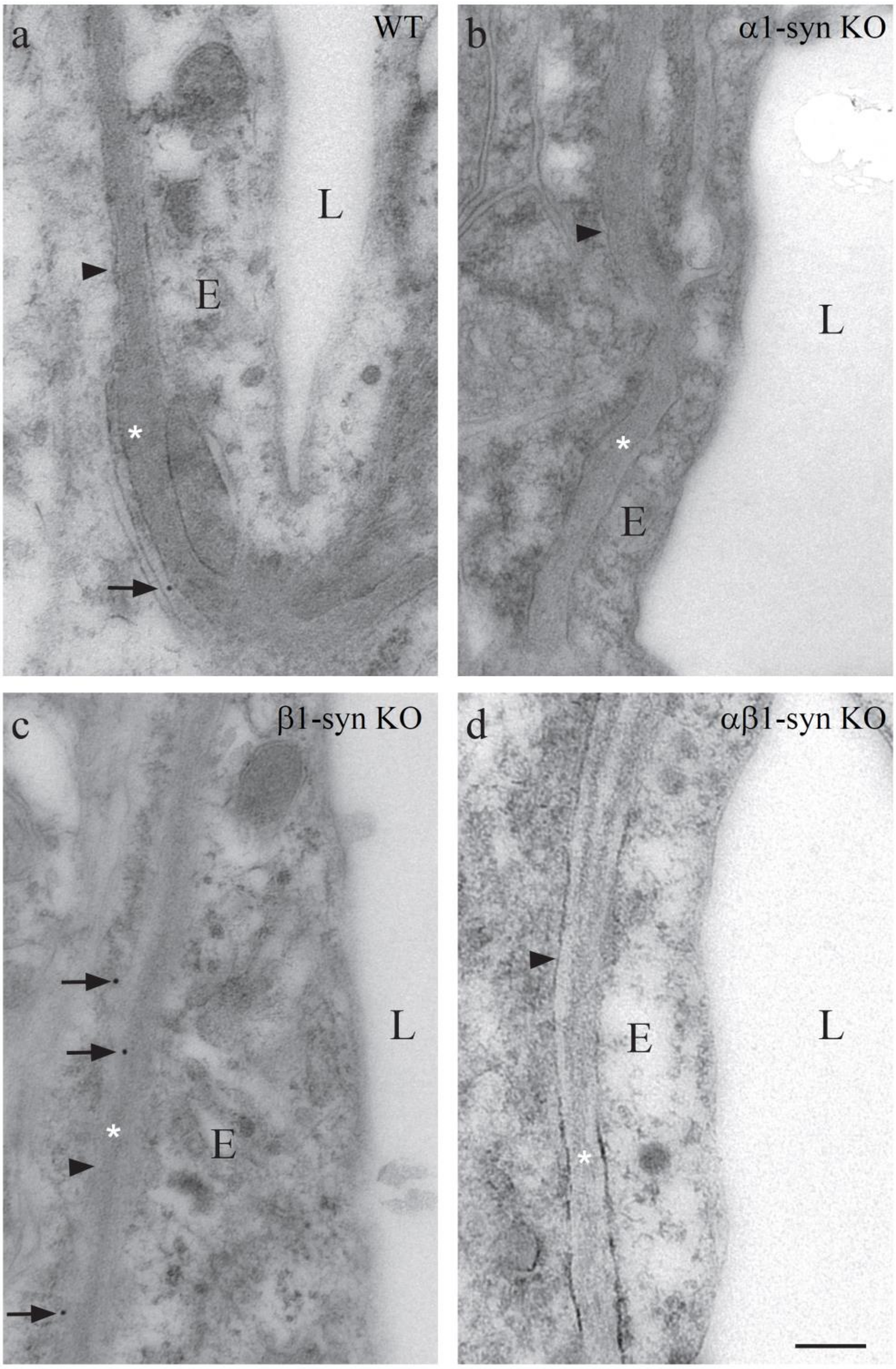

Fig. S7 Increased labeling of α1-syn in β1-syn KO retina. Confocal immunofluorescent images showing α1-syn (in red) labeling in retinae of WT (top panels) and β1-syn KO (bottom panels) mice. In mice lacking β1-syn, there was an increased labeling of α1-syn when compared to WT. Endothelial marker lectin is shown in green. Nuclear staining is shown in blue. Scale bars=20μm

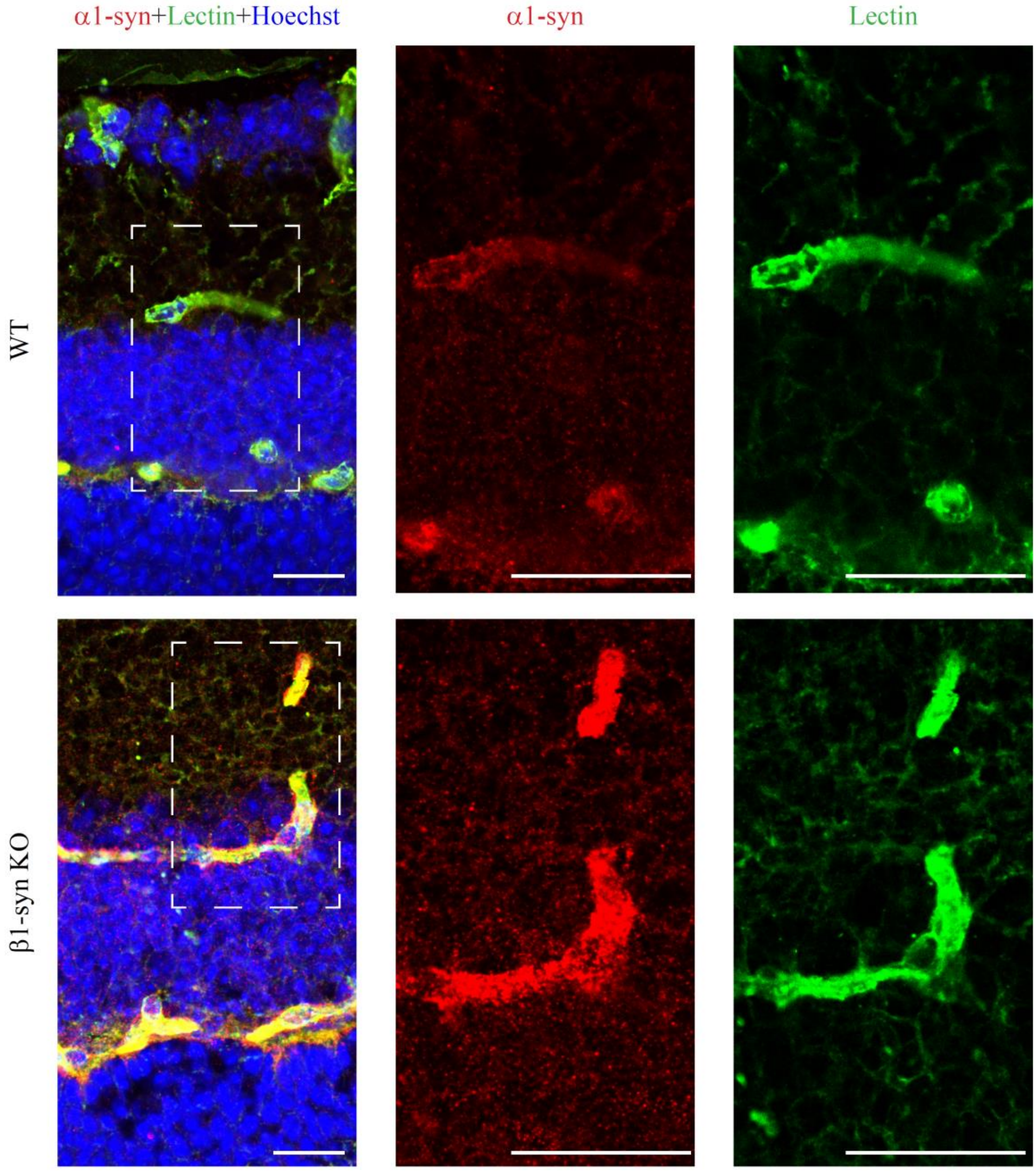

Supplement: Supplementary file 1 — Additional file 1 Figure S1 qPCR analysis of total RNA extracted from retinae using primers specific for Aqp4. No statistically significant difference in total Aqp4 gene expression was observed between the WT, α1-syn KO and αβ1-syn KO mice (n = 4 for each group). Statistics: Mann-Whitney U-test. Tbp was used as the normalization gene. Data shown as mean ± SEM. Figure S2 Control experiment for AQP4 antibody specificity. AQP4 antibody specificity was confirmed by incubating the retina samples from AQP4 KO and WT mice with the antibody. Confocal image showing a lack of specific labeling confirming the specificity of the antibody (panels a and b). GCL-ganglion cell layer; IPL-inner plexiform layer; INL-inner nuclear layer; OPL-outer plexiform layer; ONL-outer nuclear layer. Scale bars = 20 μm. Bottom: Immunogold staining also revealed a lack gold particles signaling AQP4 (bottom panel). Scale bar = 200 nm. Figure S3 Electron micrographs from an AQP4 KO mouse retina section subjected to immunogold procedure using the anti-AQP4 antibody. There is no AQP4 immunogold labeling at the perivascular Müller cell endfeet in the three retinal vascular layers (a-c). GCL-ganglion cell layer; IPL-inner plexiform layer; OPL-outer plexiform layer; L-lumen; E-endothelium; P-pericyte; * = basement membrane. The arrowheads point to the endfoot domain facing the blood vessel. Scale bar = 1 μm. Figure S4 Perivascular AQP4 localization is retained in retina of α1-syn KO mice. Confocal images showing AQP4 (in red) immunofluorescence labeling in WT and in α1-syn KO mice along with the endothelial marker lectin (in green). AQP4 is concentrated at the perivascular region in WT animals (arrows in panels a and c). AQP4 labeling is retained in mice lacking α1-syn (arrows in panels b and d). Nuclear staining is shown in blue. GCL-ganglion cell layer; IPL-inner plexiform layer; INL-inner nuclear layer; OPL-outer plexiform layer; ONL-outer nuclear layer. Scale bars = 20 μm. Immunoblot showing AQP4 exp [file 13041_2020_581_MOESM1_ESM.pdf]
