# Supplementary figures and images for: Functional specialization of retinal Müller cell endfeet depends on an interplay between two syntrophin isoforms
Source: Mol Brain. 2020 Mar 16;13:40. doi: 10.1186/s13041-020-00581-w (PMC7074989; doi:10.1186/s13041-020-00581-w)

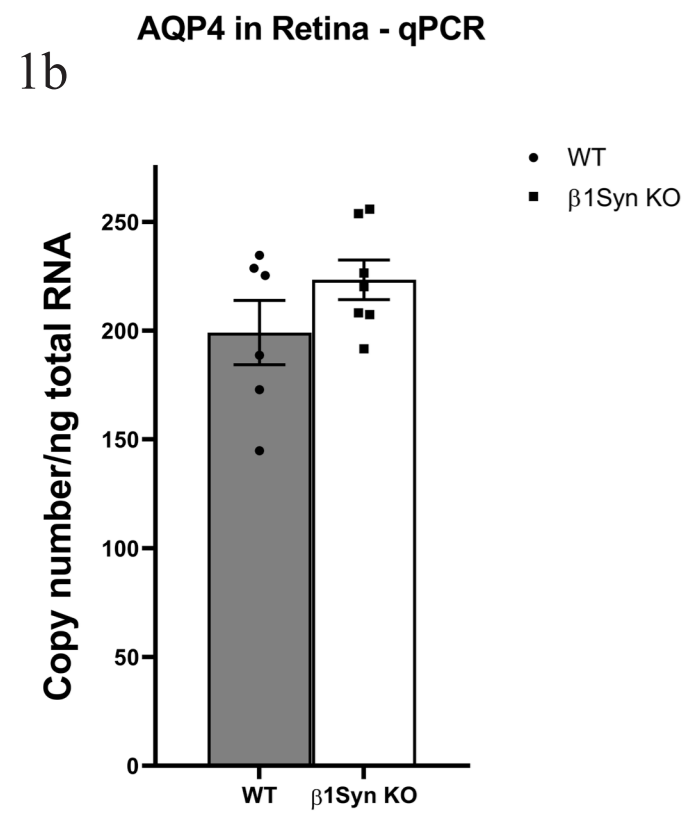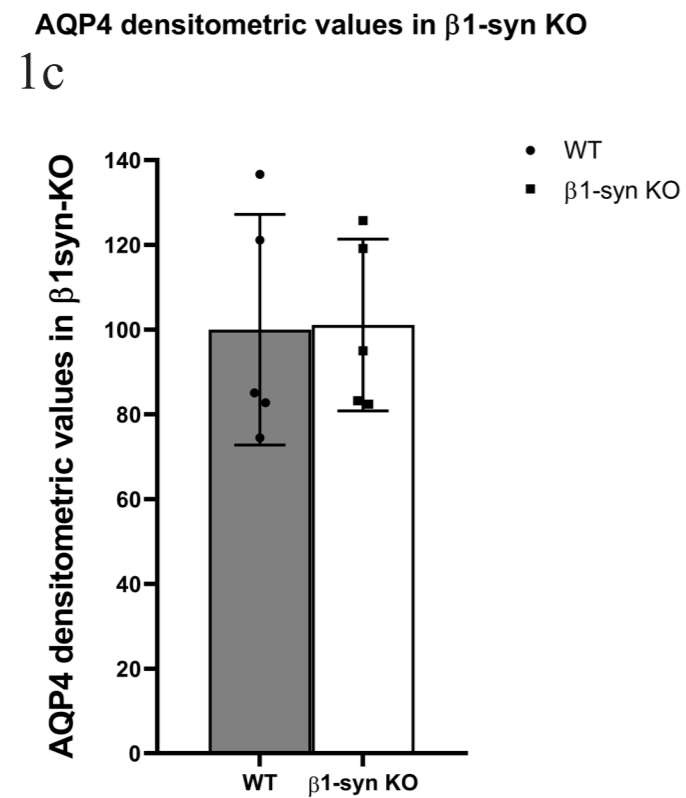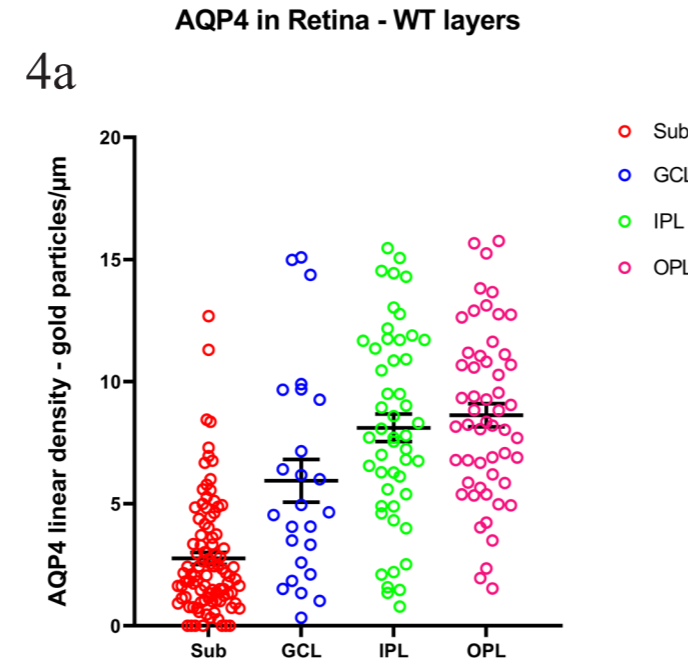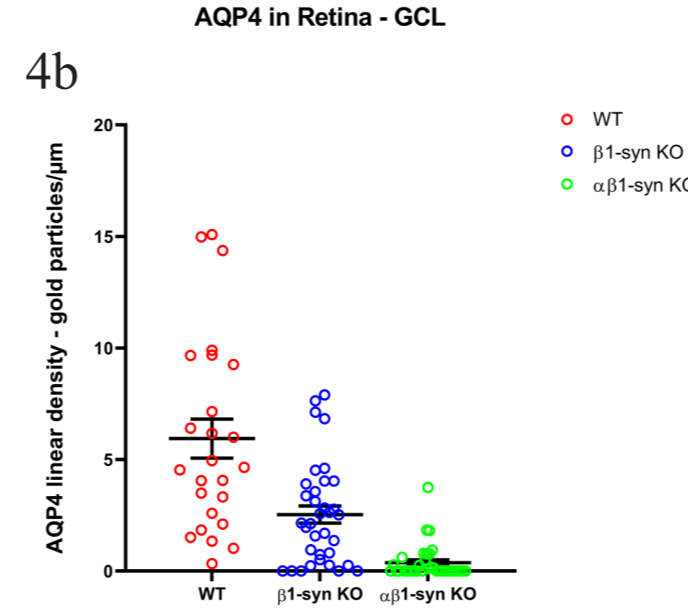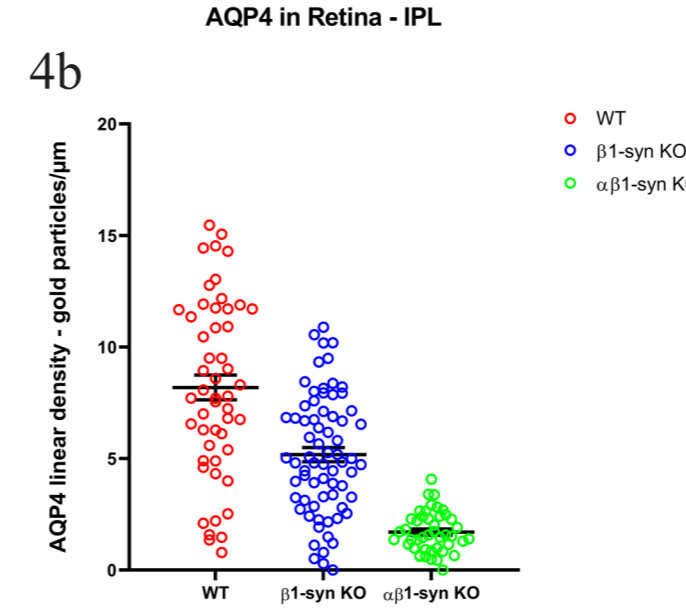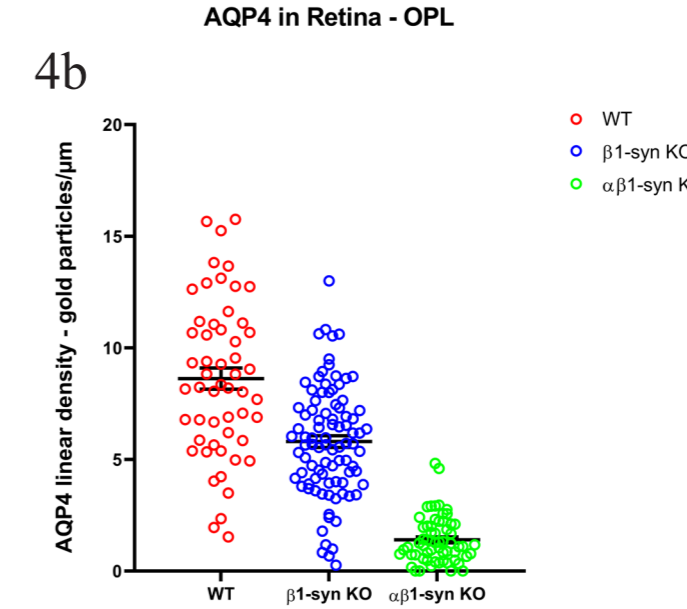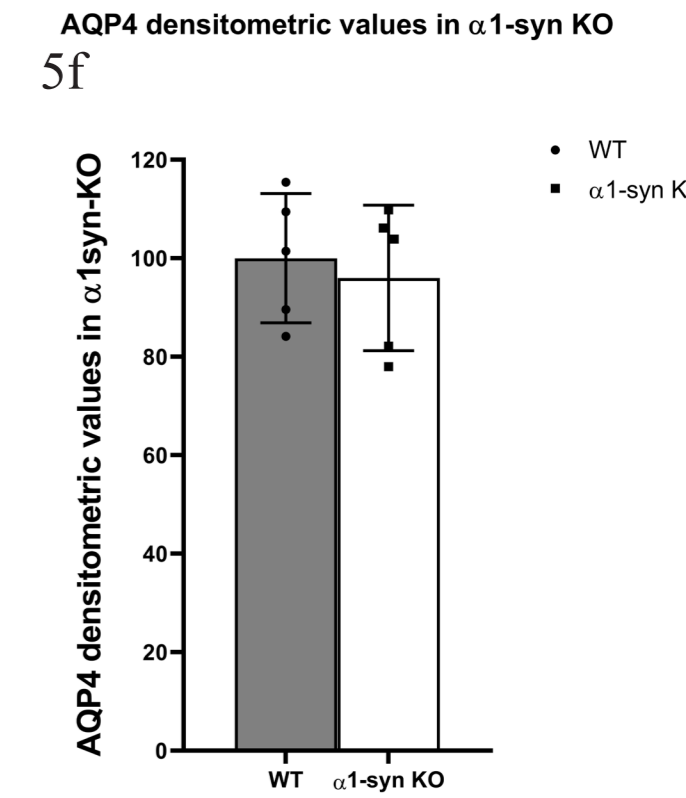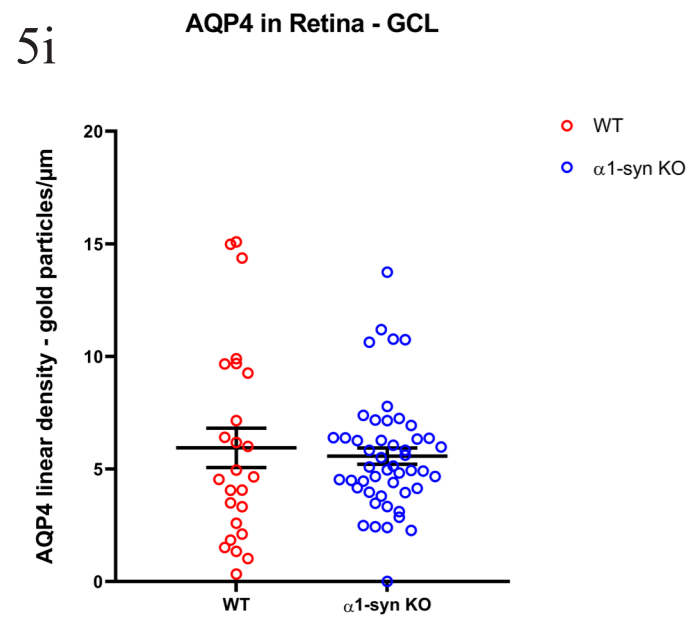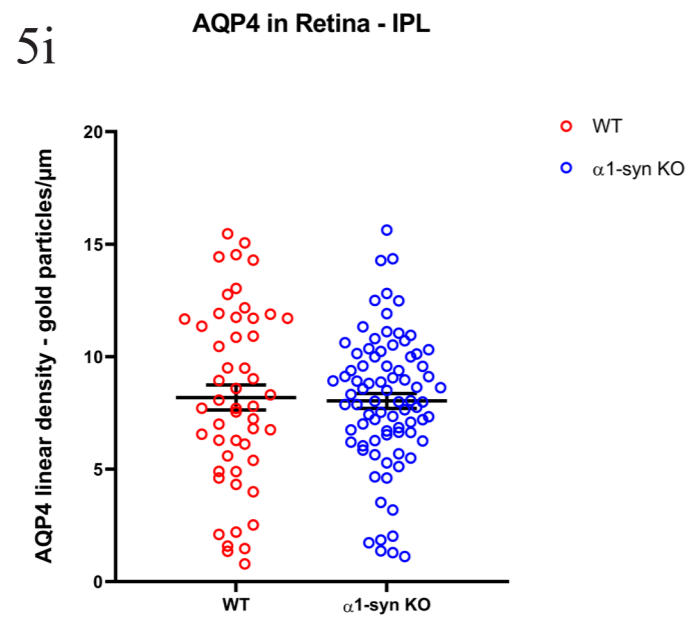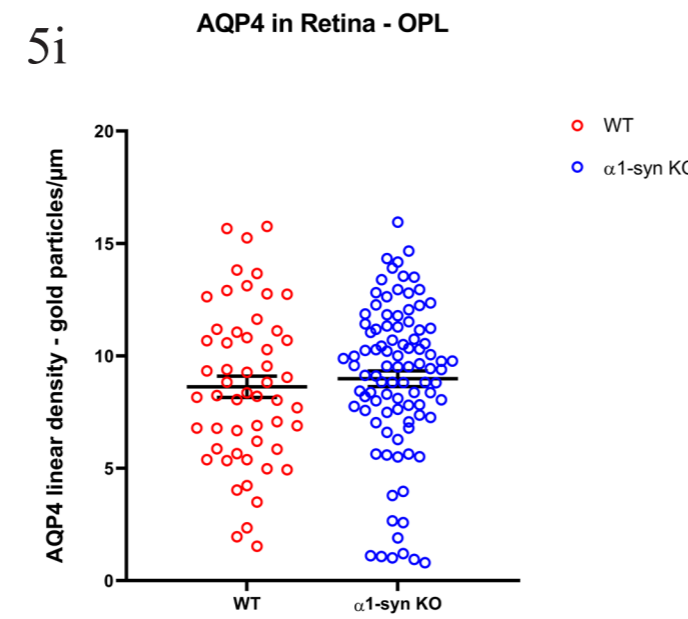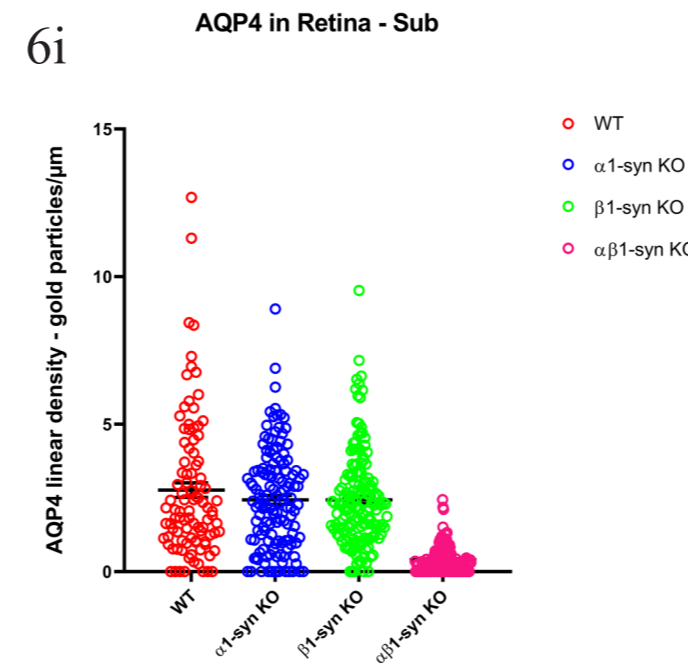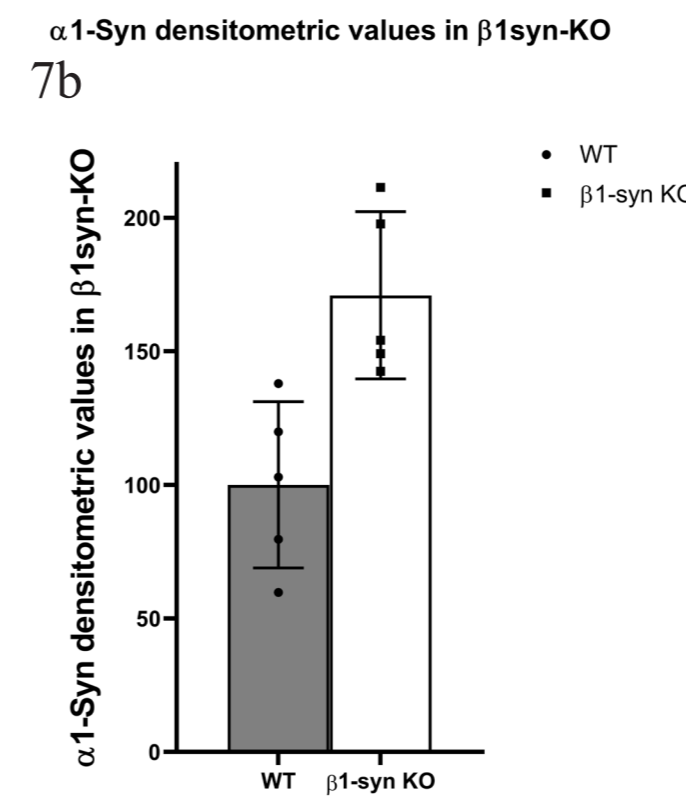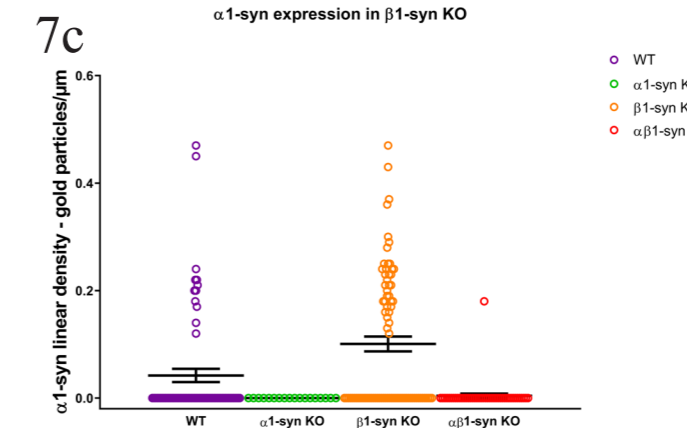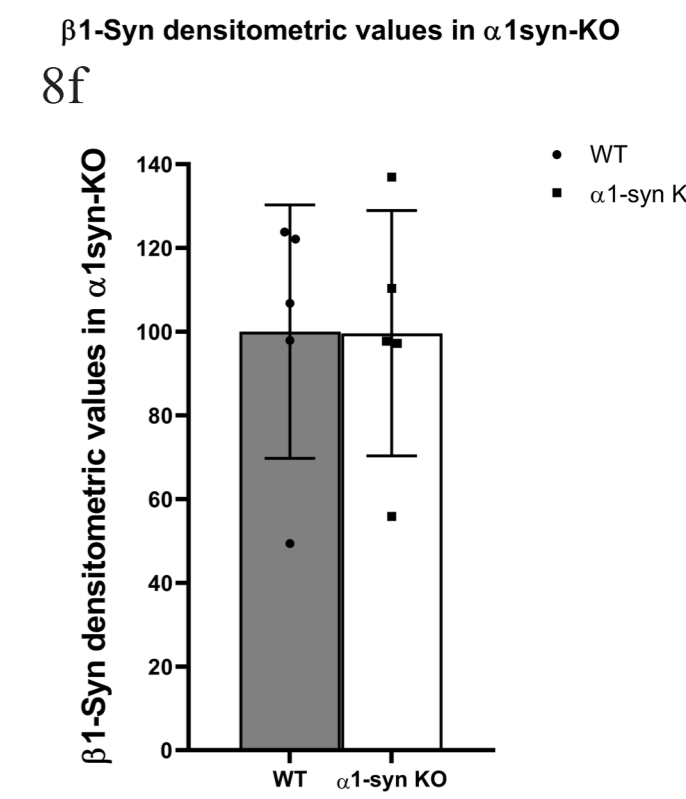

Supplement: Supplementary file 2 — Additional file 2. [file 13041_2020_581_MOESM2_ESM.pdf]
